# Supplementary material for: Synthesis of Temporin-SHa Retro Analogs with Lysine Addition/Substitution and Antibiotic Conjugation to Enhance Antibacterial, Antifungal, and Anticancer Activities
Source: Antibiotics (Basel). 2024 Dec 13;13(12):1213. doi: 10.3390/antibiotics13121213 (PMC11672801; doi:10.3390/antibiotics13121213)
Supplement: Supplementary file 1 [file antibiotics-13-01213-s001.zip › antibiotics-3326711-supplementary.pdf]

Synthesis of Temporin-SHa Retro Analog with Lysine Addition/Substitution and Antibiotic Conjugation to Enhance Antibacterial, Antifungal, and Anticancer Activities

Shahzad Nazir [1], Arif Iftikhar Khan [1], Rukesh Maharjan [1], Sadiq Noor Khan [1], Muhammad Adnan Akram [1], Marc Maresca [2,*], Farooq-Ahmad Khan [1] and Farzana Shaheen [1,*]

[1] Third World Center for Science and Technology, International Center for Chemical and Biological Sciences, University of Karachi, Karachi 75270, Pakistan; shahzad.nazir@iccs.edu (S.N.); arififtikhar@iccs.edu (A.I.K.); rukesh.maharjan@iccs.edu (R.M.); sadiqnoorkhan172@gmail.com (S.N.K.); adnan.akram@iccs.edu (M.A.A.); farooq.khan@iccs.edu (F.-A.K.)

[2] Aix Marseille Univ, CNRS, Centrale Med, ISM2, 13013 Marseille, France

* Correspondence: m.maresca@univ-amu.fr (M.M.); farzana-shaheen@iccs.edu (F.S.); Tel.: +33-0413945609 (M.M.); +92-21111222292 (F.S.)

Table of contents

#	Content	Page no.
Figure S1	UPLC Profile of NST-2 peptide (**1**)	3
Table S1	NMR (600 MHz, DMSO-d_6) data of NST-2 peptide (**1**)	4–7
Figure S2	UPLC Profile of RNST-2 peptide (**2**)	8
Figure S3	LR-ESI-MS spectrum of RNST-2 peptide (**2**)	9
Figure S4	HR-ESI-MS spectrum of RNST-2 peptide (**2**)	10
Figure S5	^{1}H-NMR spectrum (600 MHz, DMSO-d_6) of RNST-2 peptide (**2**)	11
Table S2	NMR (600 MHz, DMSO-d_6) Data of RNST-2 peptide (**2**)	12–15
Figure S6	UPLC Profile of RSP-1 peptide (**3**)	17
Figure S7	LR-ESI-MS spectrum of RSP-1 peptide (**3**)	18
Figure S8	HR-ESI-MS Spectrum of RSP-1 peptide (**3**)	19
Figure S9	^{1}H-NMR spectrum (800 MHz, DMSO-d_6) of RSP-1 peptide (**3**)	20
Table S3	NMR (800 MHz, DMSO-d_6) data of RSP-1 peptide (**3**)	21–24
Figure S10	UPLC Profile of RLFP-1 peptide (**4**)	25
Figure S11	LR-ESI-MS spectrum of RLFP-1 peptide (**4**)	26
Figure S12	HR-ESI-MS Spectrum of RLFP-1 peptide (**4**)	27
Figure S13	^{1}H-NMR spectrum (600 MHz, DMSO-d_6) of RLFP-1 peptide (**4**)	28
Table S4	NMR (600 MHz, DMSO-d_6) data of RLFP-1 peptide (**4**)	29–33
Figure S14	UPLC Profile of RLFP-2 peptide (**5**)	35
Figure S15	LR-ESI-MS spectrum of RLFP-2 peptide (**5**)	36
Figure S16	^{1}H-NMR spectrum (800 MHz, DMSO-d_6) of RLFP-2 peptide (**5**)	37
Table S5	NMR (800 MHz, DMSO-d_6) data of RLFP-2 peptide (**5**)	38–42

Figure S17	UPLC Profile of RLFP-3 peptide (**6**)	43
Figure S18	LR-ESI-MS spectrum of RLFP-3 peptide (**6**)	44
Figure S19	HR-ESI-MS Spectrum of RLFP-3 peptide (**6**)	46
Figure S20	^{1}H-NMR spectrum(800 MHz, DMSO-d_6) of RLFP-3 peptide (**6**)	45
Table S6	NMR (800 MHz, DMSO-d_6) data of RLFP-3 peptide (**6**)	47–51
Figure S21	UPLC Profile of RSP-4 peptide (**7**)	52
Figure S22	LR-ESI-MS spectrum of RSP-4 peptide (**7**)	53
Figure S23	HR-ESI-MS Spectrum of RSP-4 peptide (**7**)	54
Figure S24	^{1}H-NMR spectrum(600 MHz, DMSO-d_6) of RSP-4 peptide (**7**)	55
Table S7	NMR (600 MHz, DMSO-d_6) data of RSP-4 peptide (**7**)	56–59
Figure S25	UPLC Profile of RLFP-4 peptide (**8**)	60
Figure S26	LR-ESI-MS spectrum of RLFP-4 peptide (**8**)	61
Figure S27	HR-ESI-MS Spectrum of RLFP-4 peptide (**8**)	62
Figure S28	^{1}H-NMR spectrum(600 MHz, DMSO-d_6) of RLFP-4 peptide (**8**)	63
Table S8	NMR (600 MHz, DMSO-d_6) data of RLFP-4 peptide (**8**)	64–68
Figure S29	UV-Vis spectra of retro analogues RNST-2-(**2**), RSP-1 peptide (**3**), RSP-4 peptide (**7**),RLFP-1 peptide (**4**), RLFP-2 peptide (**5**), RLFP-3 peptide (**6**) and RLFP-4 peptide (**8**)	69

Structural Studies of NST-2 peptide (**1**)

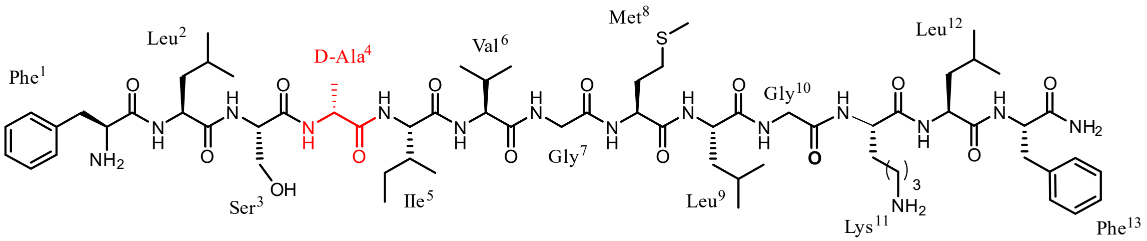

$C_{68}H_{111}N_{15}O_{14}S$
Exact Mass: 1393.82
Mol. Wt.: 1394.77

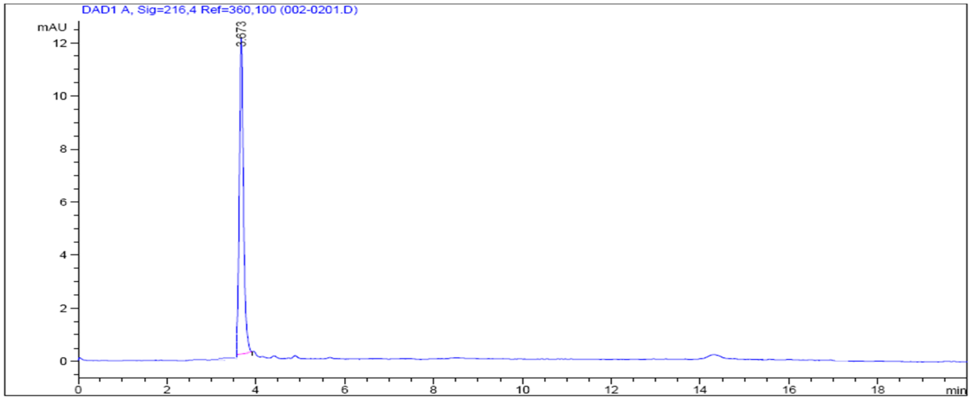

Figure S1-: UPLC profile of NST-2 peptide (**1**)

Table S1-: NMR (600 MHz, DMSO-d_6) data of NST-2 peptide (**1**) [1]

**Residue**	**Position**	NST-2 peptide (**1**)		
		**^{1}H-NMR** 600 MHz	***J*** **(Hz)**	**^{13}C-NMR** 150 MHz
Phe[1]	α	4.04 *s*		53.20
	β	2.87 *dd*, 3.07 *dd*	8.2, 14.2 & 4.38, 14.1	37.00
	1			134.7
	2-6	(7.22-7.25) *m*		126.1-129.0
	CO			167.7
	NH_2	3.4		
*Leu[2]	α	4.48 *dd*	8.9, 14.3	51.0
	β	(1.47-1.51) *m*		41.0
	γ	(1.61-1.04) *m*		24.2
	$δ_1CH_3$	(0.75-0.80) *m*		21.5,
	$δ_2$ CH_3	(0.80-0.90) *m*		23.0
	CO			171.3
	NH	8.64 *d*	8.22	
Ser[3]	CH	4.27 *dd*	5.3, 12.5	55.1
	CH2	3.60 *dd*, 3.54 *dd*	4.32, 10.2	61.7
	CO			169.4
	NH	8.19 *d*	7.56	
	OH	5.02 *s*		

Ala[4]	α	4.32 *m*		48.3
	β	1.18 *d*	6.9	18.4
	CO			172.4
	NH	7.90 *m*		
Ile[5]	α	4.24 *m*		56.7
	β	1.46 *m*		26.6
	γ	1.69 *m*		36.5
	δ1	(0.76-0.77) *m*		14.6
	δ2	(0.76-0.77) *m*		10.9
	CO			171.1
	NH	7.92 *m*		
Val[6]	α	4.10 *t*	7.5	58.0
	β	(1.93-1.97) *m*		30.2
	γ	(0.82-0.83) *m*		18.9, 19.2
	CO			171.2
	NH	7.86 *m*		
Gly[7]	α	3.63 *d* , 3.80 *d*	5.5, 5.5	41.9
	CO			168.7
	NH	8.10 *m*		
Met[8]	α	4.*34 m*		52.2
	β	(1.88-1.91) *m*		31.8
	γ	2.40 *m*		29.4

	δ	1.96 *q*	6.8	15.3
	CO			168.8
	NH	7.86 *m*		
*Leu[9]	α	4.20 *q*	8.5, 14.4	51.35
	β	(1.47-1.51) *m*		40.6
	γ	(1.61-1.04) *m*		24.0
	$δ_1CH_3$	(0.75-0.80) *m*		21.6
	$δ_2$ CH_3	(0.80-0.90) *m*		22.9
	CO			171.1
	NH	7.98 *d*	7.6	
Gly[10]	α	3.82 *d*, 3.68 *d*	5.5, 5.5	42.1
	CO			171.2
	NH	8.10 *m*		
Lys[11]	α	4.24 *m*		51.1
	β	(1.40-1.47) *m*		31.30
	γ	1.22 *m*		22.17
	δ	1.50 *m*		26.64
	ϕ	(2.71, 2.72) *m*		38.79
	CO			167.6
	NH	8.06 *d*	7.56	
	NH_2			bs
*Leu[12]	α	4.22 *m*		51.4

	β	(1.47-1.51) *m*		40.4
	γ	(1.61-1.04) *m*		24.0
	$\delta_1 CH_3$	(0.75-0.80) *m*		21.5
	δ_2 CH_3	(0.80-0.90) *m*		23.2
	CO			171.07
	NH	8.02 *d*	7.72	
Phe^{13}	α	4.41 *dd*	8.3, 13.4	53.5
	β	2.82 *m*, 2.99 *m*		37.6
	1			137.7
	2-6	(7.22-7.25) *m*		126.1-129.0
	CO			170.76
	NH	7.76 *d*	8.16	
	NH_2	7.71 *s*		

Structural Studies of RNST-2 peptide (**2**)

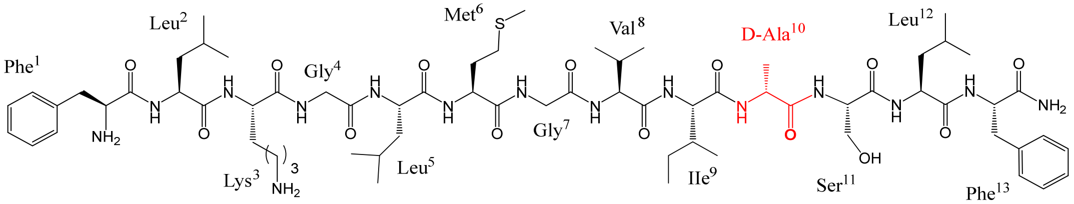

$C_{68}H_{111}N_{15}O_{14}S$
Exact Mass: 1393.82
Mol. Wt.: 1394.77

```
Data File C:\CHEM32\1\DATA\210 LAB\210 LAB 2022-12-30 12-16-46\089-1001.D
Sample Name: RNST-2

=====================================================================
Acq. Operator   : JUNAID                        Seq. Line :  10
Acq. Instrument : UPLC 4                         Location : Vial 89
Injection Date  : 12/30/2022 3:55:43 PM               Inj :   1
                                               Inj Volume : 5.000 µl
Sequence File   : C:\Chem32\1\DATA\210 LAB\210 LAB 2022-12-30 12-16-46\210 LAB.S
Method          : C:\CHEM32\1\DATA\210 LAB\210 LAB 2022-12-30 12-16-46\210 LAB.M (Sequence
                  Method)
Last changed    : 12/30/2022 12:16:35 PM by JUNAID
Method Info     : column: C-4 (4.6X250mm) 5UM 300A MN
```

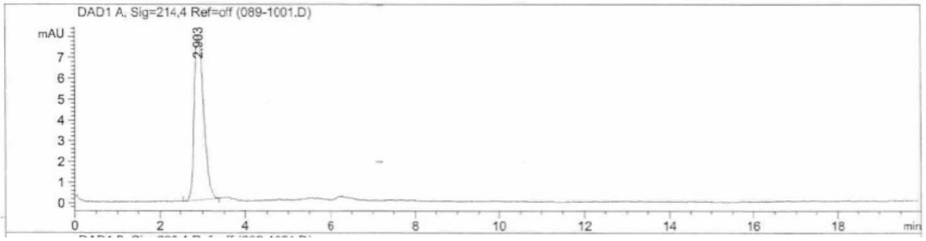

Figure S2: UPLC Profile of RNST-2 peptide (**2**)

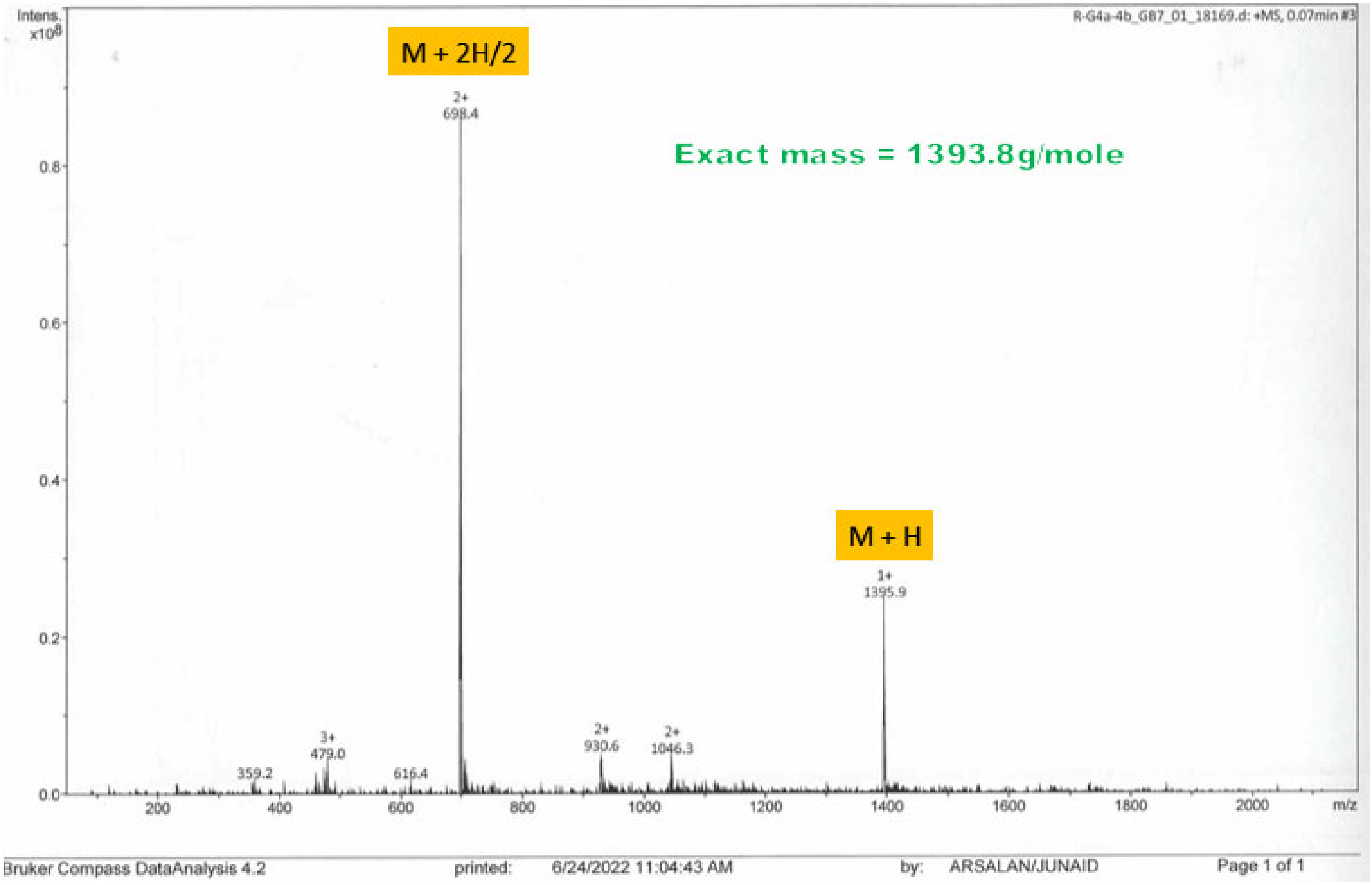

Figure S3: LR-ESI-MS spectrum of RNST-2 peptide (**2**)

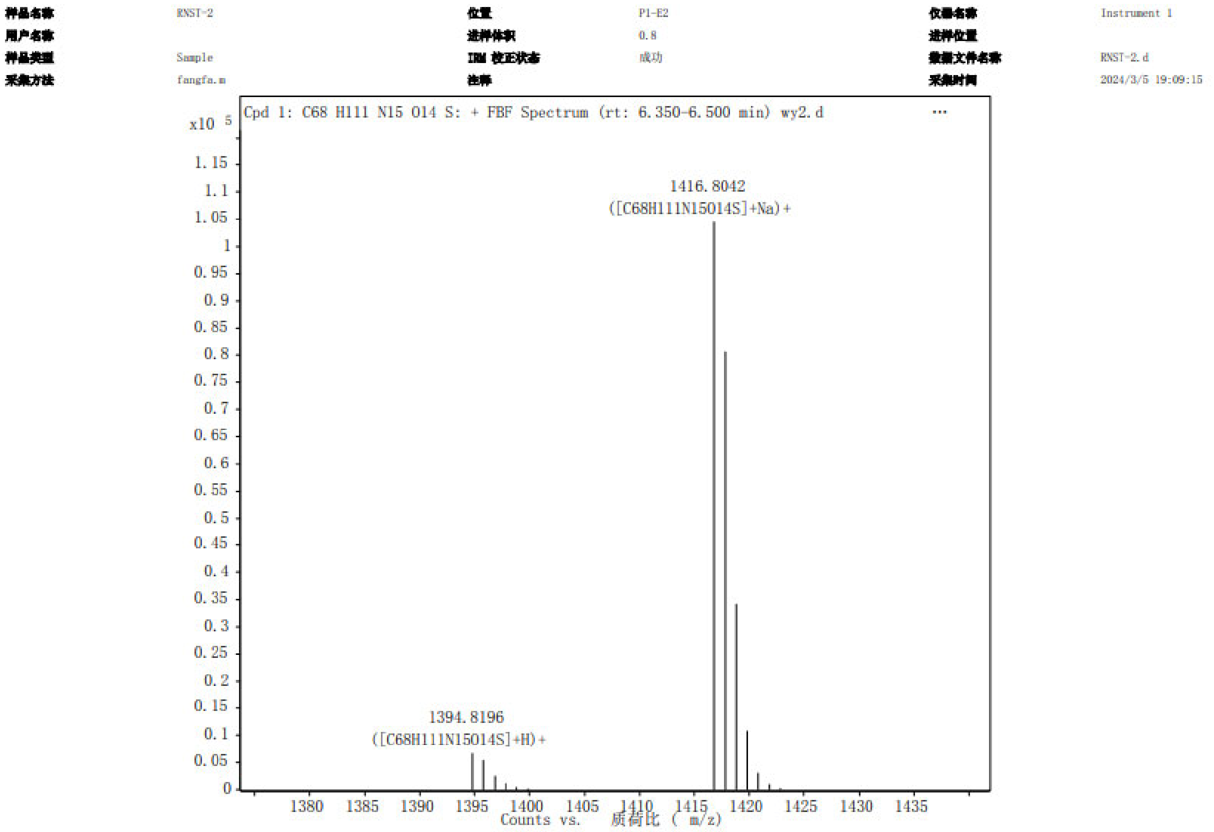

Figure S4: HR-ESI-MS spectrum of RNST-2 peptide (**2**)

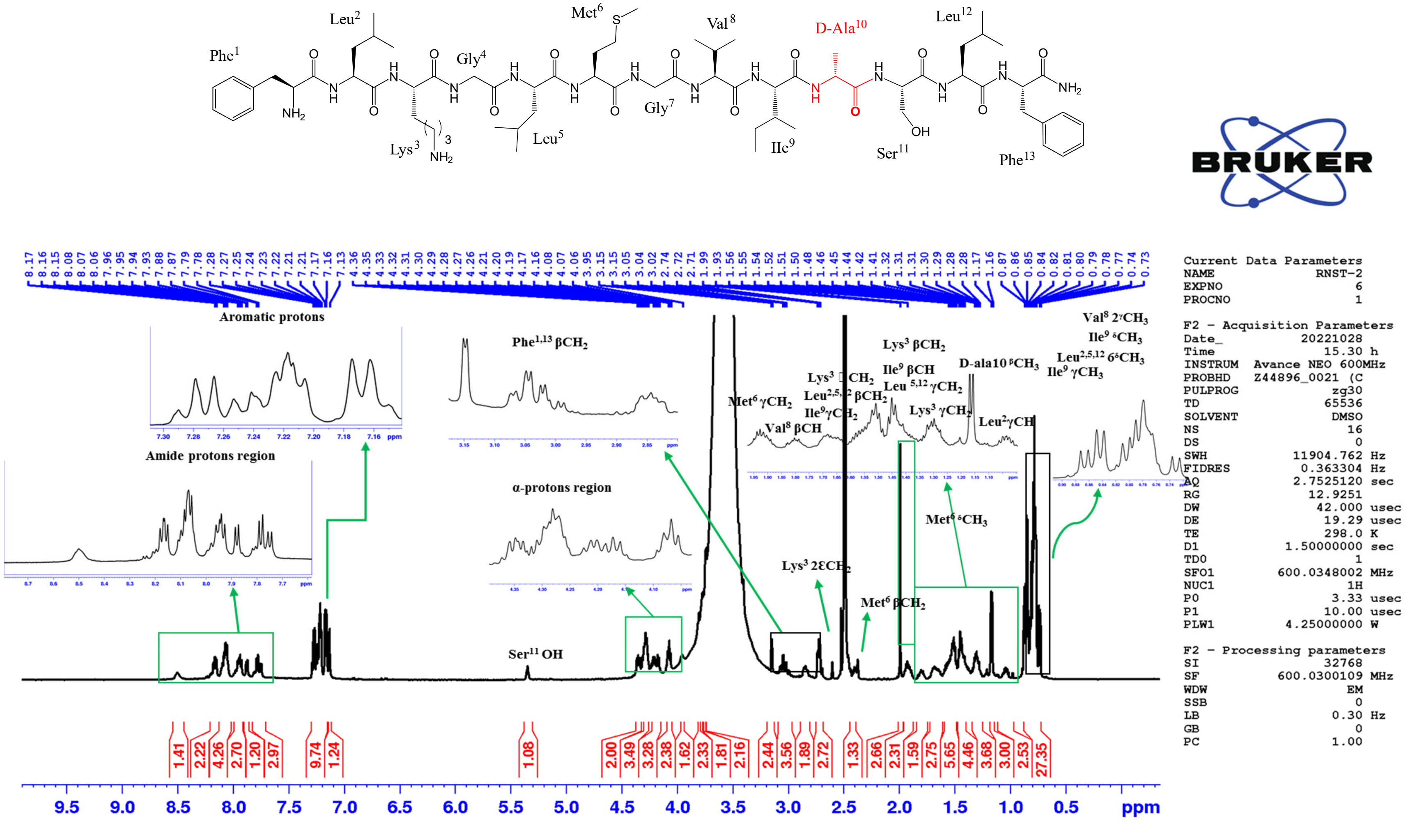

Figure S5: ^{1}H-NMR spectrum (600 MHz, DMSO-d_6) of RNST-2 peptide (**2**)

Table S2: NMR (600 MHz, DMSO-d_6) Data of RNST-2 peptide (**2**)

Residue	Position	RNST-2 peptide (**2**)		
		**^{1}H-NMR** 600 MHz	***J*** **(Hz)**	**^{13}C-NMR** 150 MHz
L-Phe[1]	α	3.94 *b*		52.2
	β	2.85, 3.04 *td*	5.28,	37.4
	1			137.9
	2-6	(7.16-7.27) *m*		127.3-128.7
	CO			173.3
	NH_2	8.49 *bs*		
L-Leu[2]	α	4.04 *m*		52.3
	β	(1.29-1.32) *m*		40.4
	γ	(1.45-1.40) *m*		24.0
	δ_1CH_3 a	(0.72-0.81) *m*		21.5
	δ_2CH_3 b	(0.83-0.87) *m*		23.2
	CO			171.9
	NH	7.74 *d*	8.52	
L-lys[3]	α	4.20 *m*		52.8
	β	(1.43-1.45) *m*		31.6
	γ	1.30 *m*		22.4
	δ	(1.64-1.69) *m*		23.3
	ϕ	2.72 *t*	7.5	36.4
	NH_2			
	CO			171.9

	NH	8.24 *d*	7.4	
Gly[4]	α	3.80, 3.77 *m*		42.0
	CO			170.9
	NH	7.79 *m*		
L-Leu[5]	α	4.04 *m*		54.0
	β	(1.29-132) *m*		41.0
	γ	(1.49-1.45) *m*		24.8
	δ_1	(0.72-0.81) *m*		21.7
	δ_2	(0.83-0.87) *m*		23.1
	CO			172.4
	NH	7.74 *d*	8.57	
L-Met[6]	α	4.12 *m*		54.8
	β	2.39 *m*		29.7
	γ	(1.81-1.78) *m*		31.8
	ϕ CH_3	1.99 *s*		15.4
	CO			171.0
	NH	7.86 *d*		
Gly[7]	α	3.80 , 3.77		42.1
	CO			170.1
	NH	8.08 *t*		
L-Val[8]	α	4.34 *m*		58.0
	β	*1.92 m*		30.6
	γ1	(0.83-0.87) *m*		18.4
	γ2	(0.83-0.87) *m*		19.3
	CO			171.4

	NH	7.95 *m*		
L-Ile[9]	α	4.18 *m*		54.9
	β	(1.45-1.52) *m*		26.8
	$\gamma_1 CH_3$	(0.87-0.84) *m*		14.7
	$\gamma_2 CH_2$	(1.52-1.60) *m*		34.6
	δCH_3	(0.84-0.80) *m*		11.1
	CO			171.9
	NH	8.01		
D-Ala[10]	α	4.26		48.4
	β	1.16 *d*	7.02	18.3
	CO			171.9
	NH	7.95 *m*		
L-Ser[11]	α	4.22 *m*		57.7
	β	3.60 *m*		61.9
	OH	5.3 *bs*		
	CO			170.8
	NH	8.01		
L-Leu[12]	α	4.12 *m*		52.3
	β	(1.43-1.49) *m*		40.8
	γ	(1.55-1.50) *m*		24.2
	$\gamma 1 CH_3$ a	(0.73-0.80) *m*		21.6
	$\gamma 1 CH_3$ b	(0.80-0.85) *m*		21.7
	CO			171.7
	NH	7.88 *d*	7.5	

L-Phe[13]	α	3.94 *m*		51.3
	β	3.05 *td*, 3.15 *d*	5.8, 3.44	37.4
	1			137.9
	2-6	(7.16-7.27) *m*		126.5-129.5
	CO			168.9
	NH	8.02		
	NH_2	7.12 *s*		

Structural Studies of RSP-1 peptide (**3**)

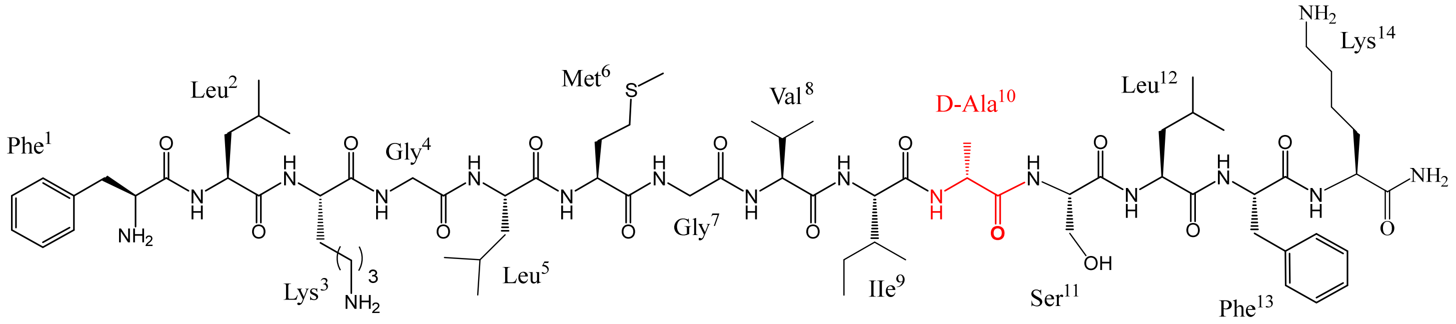

Chemical Formula: $C_{74}H_{123}N_{17}O_{15}S$
Exact Mass: 1521.911
Molecular Weight: 1522.962

Data File C:\CHEM32\1\DATA\SHAHZAD NAZIR\SHAHZAD NAZIR 2022-06-22 12-20-52\011-0201.D
Sample Name: RSP-1

===

Acq. Operator	: Junaid Analyst	Seq. Line :	2
Acq. Instrument	: UPLC-2	Location :	Vial 11
Injection Date	: 6/22/2022 1:04:19 PM	Inj :	1
		Inj Volume :	5.000 µl

Sequence File : C:\Chem32\1\DATA\SHAHZAD NAZIR\SHAHZAD NAZIR 2022-06-22 12-20-52\SHAHZAD NAZIR.S
Method : C:\CHEM32\1\DATA\SHAHZAD NAZIR\SHAHZAD NAZIR 2022-06-22 12-20-52\SHAHZAD NAZIR.M (Sequence Method)
Last changed : 6/22/2022 12:18:50 PM by Junaid Analyst
Method Info : Column: C-4 (4.6x250mm) 5um particle size,MN

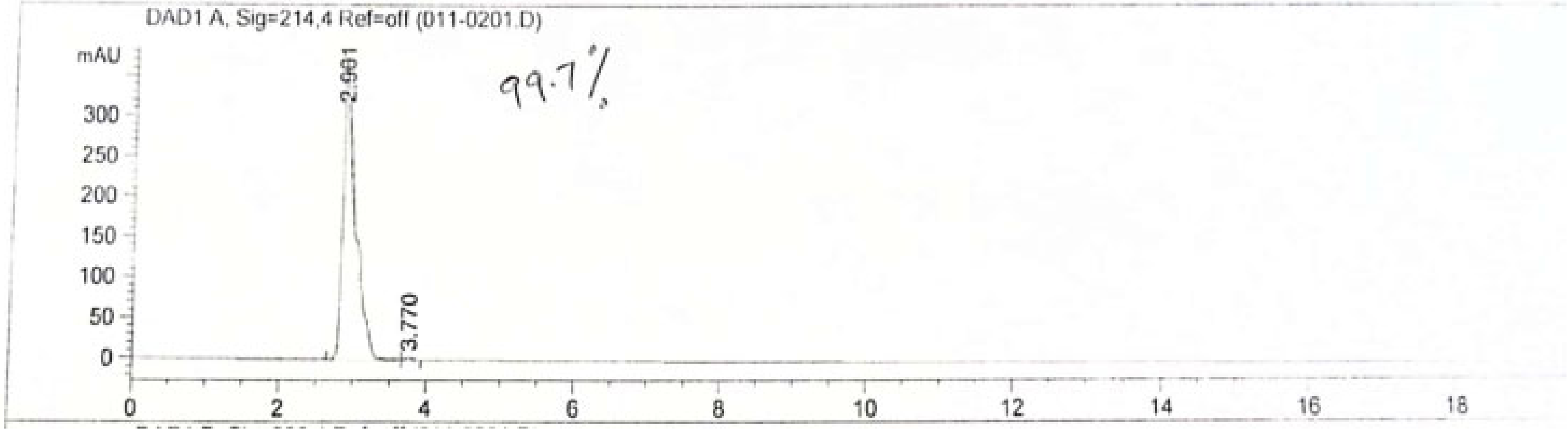

Figure S6: UPLC Profile of RSP-1 peptide **(3)**

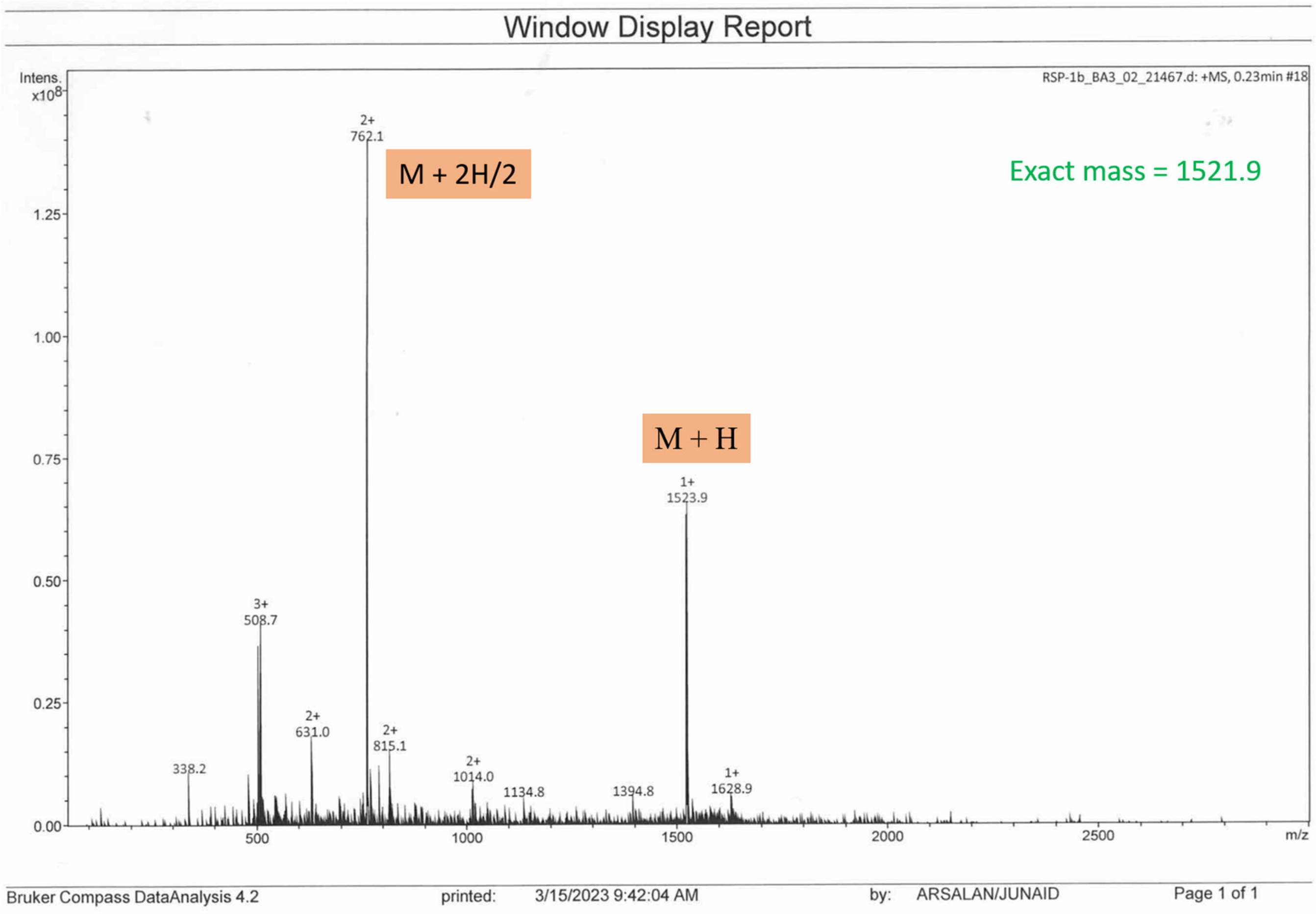

Figure S7: LR-ESI-MS spectrum of RSP-1 peptide **(3)**

样品名称	RSP-1	位置	P1-E2	仪器名称	Instrument 1
用户名称		进样体积	0.8	进样位置	
样品类型	Sample	IRM 校正状态	成功	数据文件名称	RSP-1.d
采集方法	jiachun10%-100%12min.m	注释		采集时间	2024/3/6 13:40:46

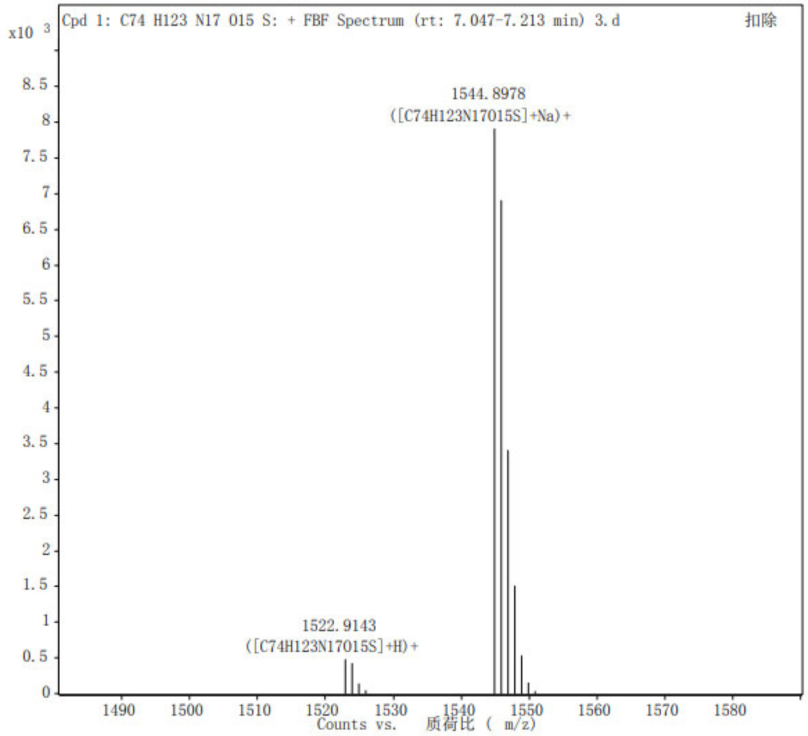

Figure S8: HR-ESI-MS Spectrum of RSP-1 peptide (**3**)

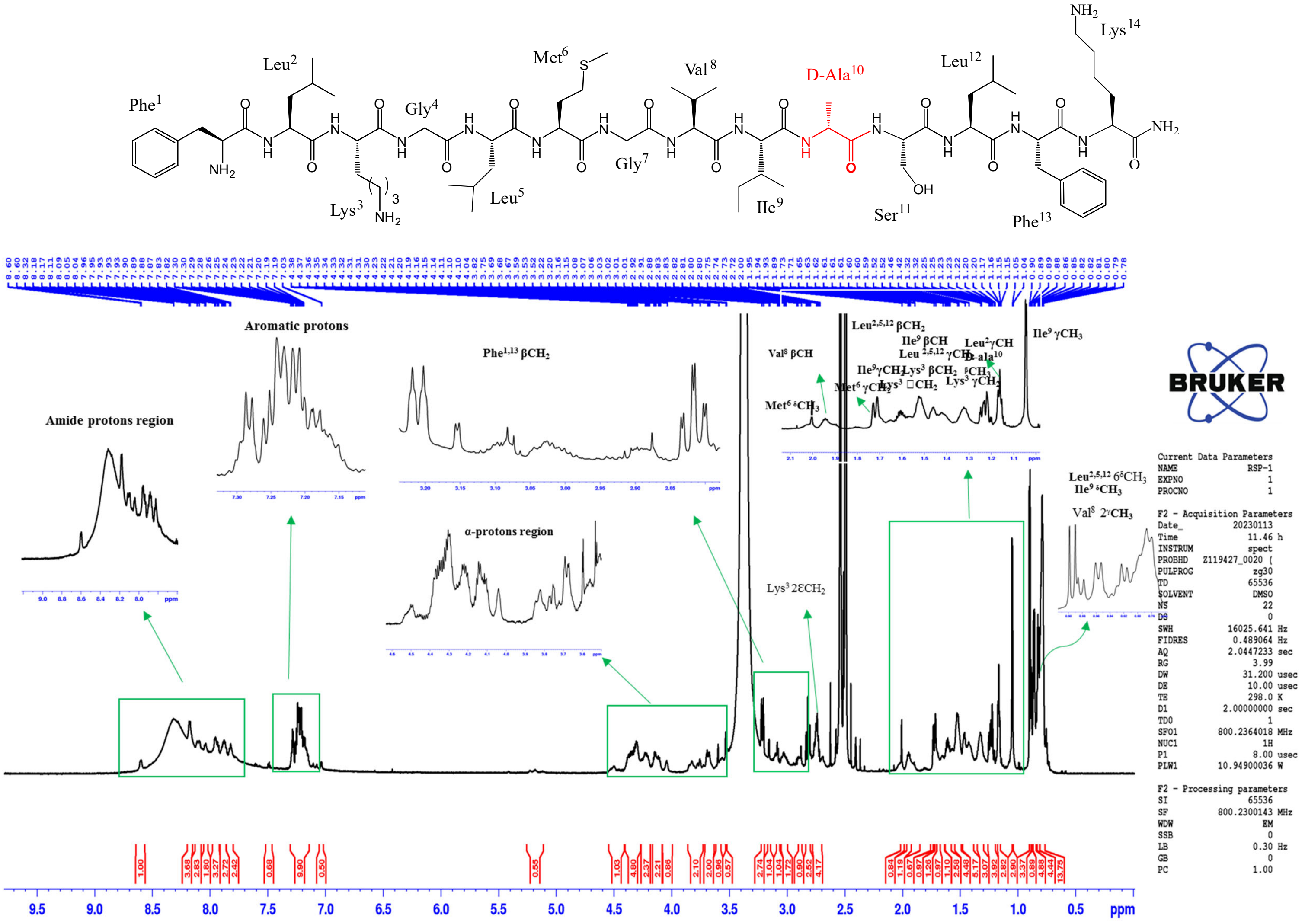

Figure S9: ^{1}H-NMR spectrum (800 MHz, DMSO-d_6) of RSP-1 peptide **(3)**

Table S3: NMR (800 MHz, DMSO-d_6) Data of RSP-1 peptide (**3**)

**Residue**	**Position**	RSP-1 peptide (**3**)		
		**^{1}H-NMR** 600 MHz	***J*** **(Hz)**	**^{13}C-NMR** 150 MHz
L-Phe[1]	α	4.04*bs*		53.3
	β	2.89 *d*, 3.03 *m*		37.4
	1			137.9
	2-6	(7.16-7.27) *m*		127.3-128.7
	CO			167.9
	NH_2			
L-Leu[2]	α	4.37 *m*		51.3
	β	(1.29-1.32) *m*		40.4
	γ	(1.45-1.40) *m*		24.0
	δ_1CH_3 a	(0.72-0.81) *m*		21.5
	δ_2CH_3 b	(0.83-0.87) *m*		23.2
	CO			171.2
	NH	8.60 *m*		
L-lys[3]	α	4.34 *m*		53.3
	β	(1.43-1.45) *m*		31.6
	γ	1.30 *m*		22.4
	δ	(1.64-1.69) *m*		23.3
	ϕ	2.74 *t*		38.5
	NH_2			
	CO			171.6

	NH	8.17 *m*	
Gly[4]	α	3.80, 3.77 *m*	42.0
	CO		168.4
	NH	8.17 *m*	
L-Leu[5]	α	4.15 *m*	57.3
	β	(1.29-132) *m*	41.0
	γ	(1.49-1.45) *m*	24.8
	δ_1	(0.72-0.81) *m*	21.7
	δ_2	(0.83-0.87) *m*	23.1
	CO		171.7
	NH	7.94 *m*	
L-Met[6]	α	4.23 *m*	52.6
	β	2.39 *m*	29.7
	γ	(1.81-1.78) *m*	31.8
	ϕ CH_3	1.99 *s*	15.4
	CO		171.6
	NH	8.18 *m*	
Gly[7]	α	3.80 *m* , 3.77 *m*	41.9
	CO		168.8
	NH	8.10 *m*	
L-Val[8]	α	4.19 *m*	57.8
	β	*1.92 m*	30.6
	γ1	(0.83-0.87) *m*	18.4
	γ2	(0.83-0.87 *)m*	19.3
	CO		168.8

	NH	7.84 *m*	
L-Ile[9]	α	4.13 *m*	51.8
	β	(1.45-1.52) *m*	26.8
	$\gamma_1 CH_3$	(0.87-0.84) *m*	14.7
	$\gamma_2\ CH_2$	(1.52-1.60) *m*	34.6
	δCH_3	(0.84-0.80) *m*	11.1
	CO		170.6
	NH	7.93 *m*	
D-Ala[10]	α	4.32 *m*	48.4
	β	1.16 *d*	18.3
	CO		171.7
	NH	8.05 *m*	
L-Ser[11]	α	4.30 *m*	51.1
	β	3.54 *m*, 3.60 *m*	61.7
	OH	5.3 *t*	
	CO		171.8
	NH	7.87 *m*	
L-Leu[12]	α	4.29 *m*	51.2
	β	(1.43-1.49) *m*	40.8
	γ	(1.55-1.50) *m*	24.2
	$\gamma 1CH_3$ a	(0.73-0.80) *m*	21.6
	$\gamma 1CH_3$ b	(0.80-0.85) *m*	21.7
	CO		171.0
	NH	7.84 *m*	

L-Phe[13]	α	4.49 *m*	53.6
	β	2.87 *m*, 3.15 *m*	37.4
	1		137.9
	2-6	(7.16-7.27) *m*	126.5-129.5
	CO		170.4
	NH	7.90 *m*	
	NH_2	7.12 *s*	
L-lys[14]	α	4.29 *m*	54.7
	β	(1.43-1.45) *m*	31.6
	γ	1.30 *m*	22.4
	δ	(1.64-1.69) *m*	23.3
	ϕ	2.73 *t*	38.7
	NH_2		
	CO		170.1
	NH	8.11 *m*	

Structural Studies of (RLFP-1) peptide (**4**)

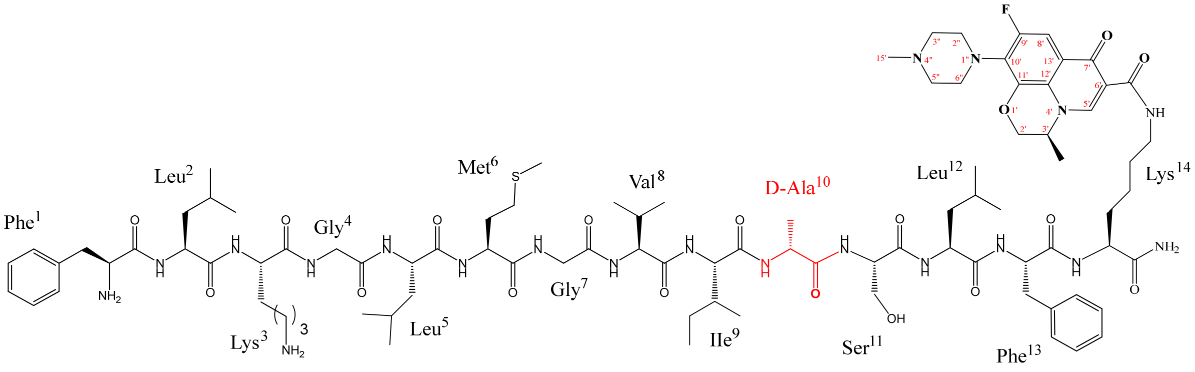

Chemical Formula: $C_{92}H_{141}FN_{20}O_{18}S$
Exact Mass: 1865.044
Molecular Weight: 1866.320

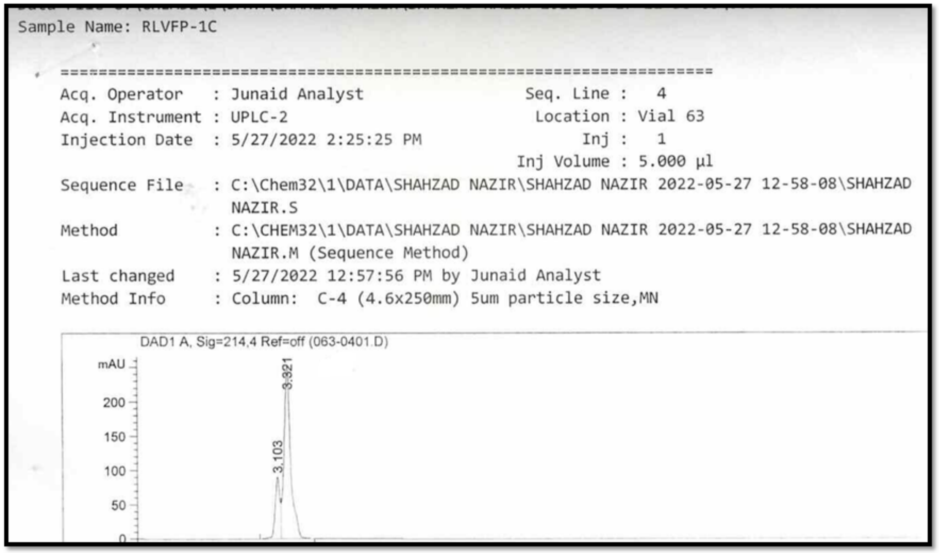

Sample Name: RLVFP-1C

===

Acq. Operator : Junaid Analyst Seq. Line : 4
Acq. Instrument : UPLC-2 Location : Vial 63
Injection Date : 5/27/2022 2:25:25 PM Inj : 1
Inj Volume : 5.000 µl
Sequence File : C:\Chem32\1\DATA\SHAHZAD NAZIR\SHAHZAD NAZIR 2022-05-27 12-58-08\SHAHZAD NAZIR.S
Method : C:\CHEM32\1\DATA\SHAHZAD NAZIR\SHAHZAD NAZIR 2022-05-27 12-58-08\SHAHZAD NAZIR.M (Sequence Method)
Last changed : 5/27/2022 12:57:56 PM by Junaid Analyst
Method Info : Column: C-4 (4.6x250mm) 5um particle size,MN

Figure S10: UPLC Profile of RLFP-1 peptide (**4**)

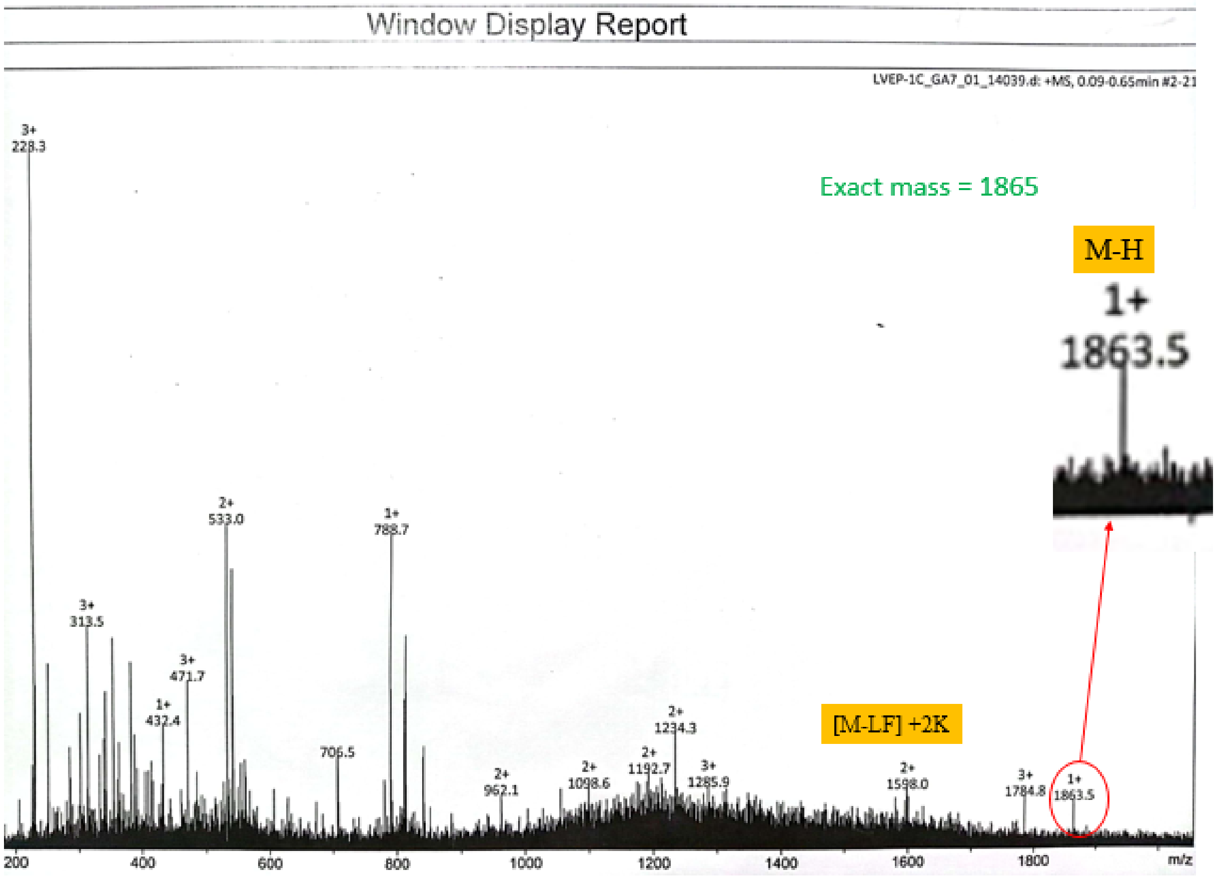

Figure S11: LR-ESI-MS spectrum of RLFP-1 peptide (**4**)

样品名称	RLVFP-1	位置	P1-E4	仪器名称	Instrument 1
用户名称		进样体积	0.8	进样位置	
样品类型	Sample	IRM 校正状态	成功	数据文件名称	RLVFP-1.d
采集方法	jiachun10%-100%12min.m	注释		采集时间	2024/3/6 14:06:11

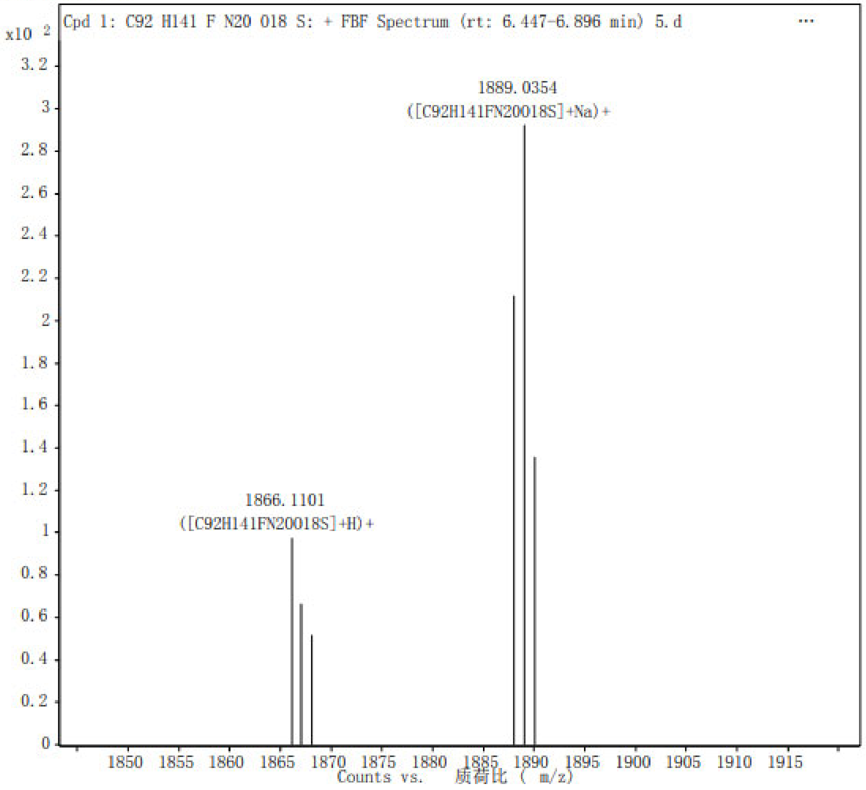

Figure S12: HR-ESI-MS Spectrum of RLFP-1 peptide **(4)**

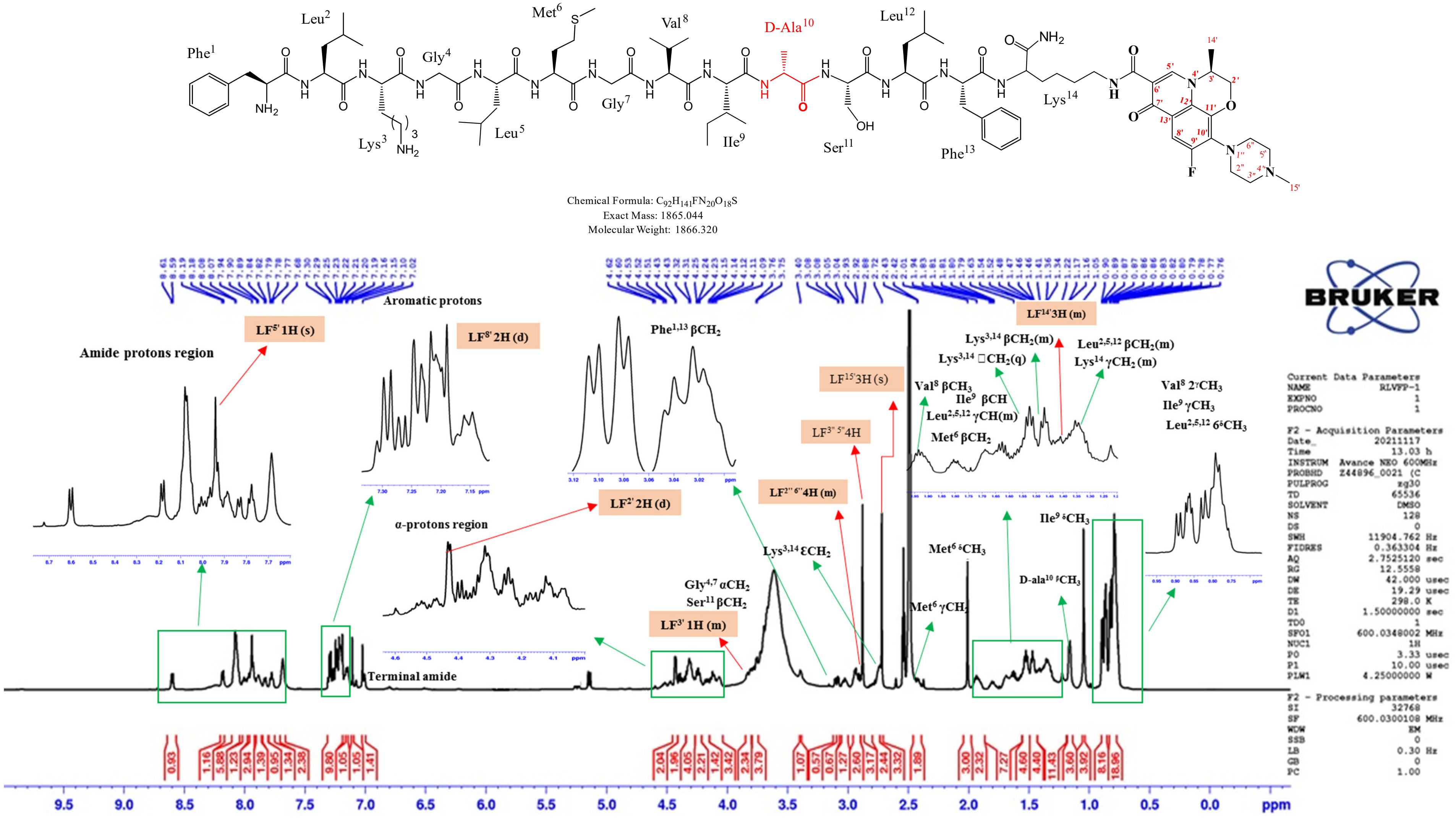

Figure S13: ^{1}H-NMR spectrum (600 MHz, DMSO-d_6) of RLFP-1 peptide (**4**)

Table S4: NMR (600 MHz, DMSO-d_6) Data of RLFP-1 peptide (**4**)

Residue	Position	RLFP-1 peptide (**4**)		
		**^{1}H-NMR** 600 MHz	***J*** **(Hz)**	**^{13}C-NMR** 150 MHz
L-Phe[1]	α	4.04 *m*		53.39
	β	(2.87-2.94)		37.2
		(3.01-3.10) *d*	4.86	
	1			137.7
	2-6	(7.00-7.30) *m*		126.5-129.3
	CO			168.0
	NH_2	8.07 *d*		
L-Leu[2]	α	4.36 *m*		51.4
	β	(1.33-1.37) *m*		41.0
	γ	(1.79-1.81) *m*		24.2
	δ_1CH_3 a	(0.75-0.81) *m*		21.8
	δ_2CH_3 b	(0.85-0.87) *m*		23.0
	CO			167.9
				171.8
	NH	8.58		
L-lys[3]	α	4.08 *m*		52.8
	β	(1.41-1.48) *m*		31.5
	γ	1.30 *m*		22.4
	δ	(1.53-1.50) *m*		24.7
	ϕ	2.73-2.79 *m*		38.9

	NH_2		
	CO		171.1
	NH	7.75	
Gly[4]	α	(3.79-3.80) *m*	42.0
	CO		168.9
	NH	8.07 *m*	
L-Leu[5]	α	4.15 *m*	51.7
	β	(1.33-137) *m*	40.8
	γ	(1.45-1.48) *m*	24.3
	δ_1	(0.72-0.81) *m*	21.7
	δ_2	(0.83-0.87) *m*	23.1
	CO		171.1
			172.3
	NH	7.75 *d*	
L-Met[6]	α	4.27 *m*	51.3
	β	(2.38-2.45) *m*	31.7
	γ	(1.80) *m*	29.7
	ϕ CH_3	2.00*s*	14.9
	CO		172.2
	NH	7.94 *d*	
Gly[7]	α	3.6-3.8	42.1
	CO		168.9
	NH	8.07	
L-Val[8]	α	4.18 *m*	58.1
	β	*1.93 m*	30.6

	γ1	(0.78-0.80) *m*		18.4
	γ2	(0.80-0.83)*m*		19.4
	CO			171.4
				169.2
	NH	7.82		
L-Ile[9]	α	4.09 *m*		57.7
	β	(1.79-1.82) *m*		34.8
	$\gamma_1 CH_3$	(1.04) *s*		27.3
	γ_2 CH_2	(1.60-1.69) *m*		31.5
	δCH_3	(0.78-0.83) *m*		11.2
	CO			173.5
	NH	8.03		
D-Ala[10]	α	4.30 *m*		48.4
	β	1.15 *d*	4.26	18.4
	CO			172.1
	NH	7.94 *m*		
L-Ser[11]	α	4.28 *m*		55.0
	β	3.72-3.83 *m*		61.8
	OH	4.27 *m*		
	CO			170.5
	NH	7.85		
L-Leu[12]	α	4.27 *m*		53.4
	β	(1.33-1.37) *m*		40.3
	γ	(1.60-1.70) *m*		24.1
	$\gamma 1CH_3$ a	(0.75-0.80) *m*		21.6

	γ1CH_3 b	(0.83-0.85) *m*		23.3
	CO			171.1
	NH	7.94 *m*		
L-Phe[13]	α	4.46 *m*		54.4
	β	2.87-2.94 *m*		37.2
		3.01-3.10 *d*	4.74	
	1			134.8
	2-6	(7.00-7.30) *m*		126.5-129.3
	CO			171.0
	NH	7.92		
L-Lys[14]	α	4.22 *m*		52.8
	β	(1.35-1.40) *m*		31.5
	γ	1.30 *m*		22.4
	δ	(1.50-1.55) *m*		26.8
	ϕ	2.78 *m*		37.3
	NH	7.68 *s*		
	CO			171.8
	NH	8.19		
Levofloxacin	2'	4.31-4.33 *d*	4.62	68.2
		4.54		
	3'	4.84		53.9
	5'	8.76 *s*		145.0
	6'			110.2
	7' (CO)			173.9
	8'	7.52		103.1

9'		157.5
10'		129.0
11'		140.7
12'		124.2
13'		122.7
14'	1.40 *m*	18.06
15'	2.84 *s*	42.4
2", 6"	3.16	53.2
3", 5"	3.47	53.4
NH_2	7.68 *bs*	
CO		167.9

Structural Studies of RLFP-2 peptide (**5**)

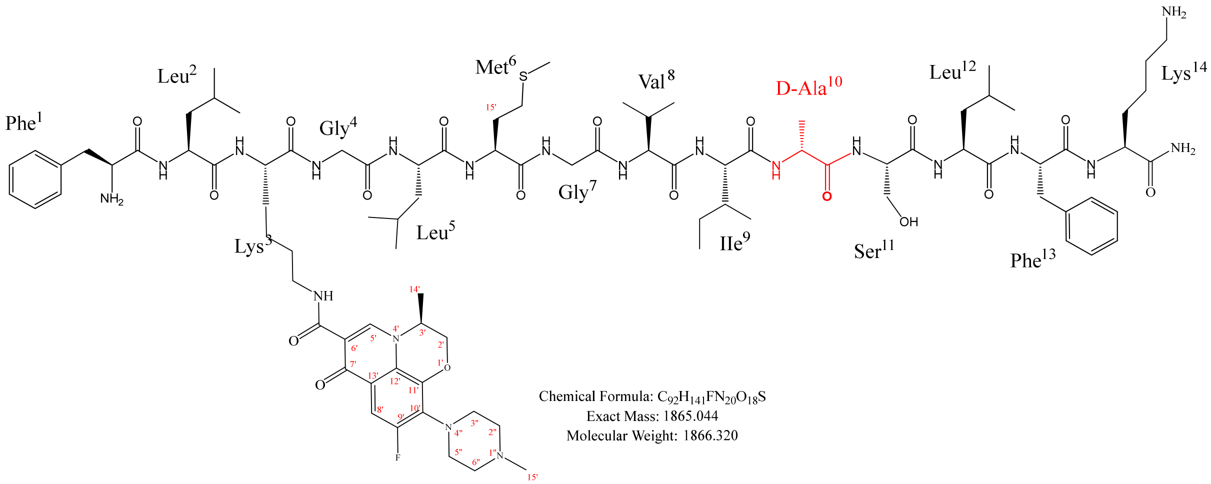

Chemical Formula: $C_{92}H_{141}FN_{20}O_{18}S$
Exact Mass: 1865.044
Molecular Weight: 1866.320

```
Data File C:\CHEM32\1\DATA\SHAHZAD NAZIR\SHAHZAD NAZIR 2022-05-27 12-58-08\062-0301.D
Sample Name: RLVFP-2F

    =====================================================================
    Acq. Operator   : Junaid Analyst                  Seq. Line :   3
    Acq. Instrument : UPLC-2                           Location : Vial 62
    Injection Date  : 5/27/2022 2:03:31 PM                  Inj :   1
                                                    Inj Volume : 5.000 µl
    Sequence File   : C:\Chem32\1\DATA\SHAHZAD NAZIR\SHAHZAD NAZIR 2022-05-27 12-58-08\SHAHZAD
                      NAZIR.S
    Method          : C:\CHEM32\1\DATA\SHAHZAD NAZIR\SHAHZAD NAZIR 2022-05-27 12-58-08\SHAHZAD
                      NAZIR.M (Sequence Method)
    Last changed    : 5/27/2022 12:57:56 PM by Junaid Analyst
    Method Info     : Column:  C-4 (4.6x250mm) 5um particle size,MN
```

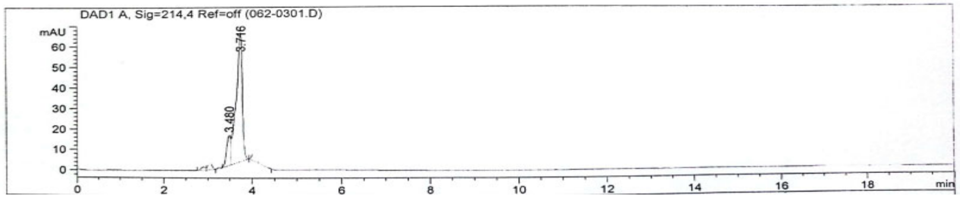

Figure S14: UPLC Profile of RLFP-2 peptide **(5)**

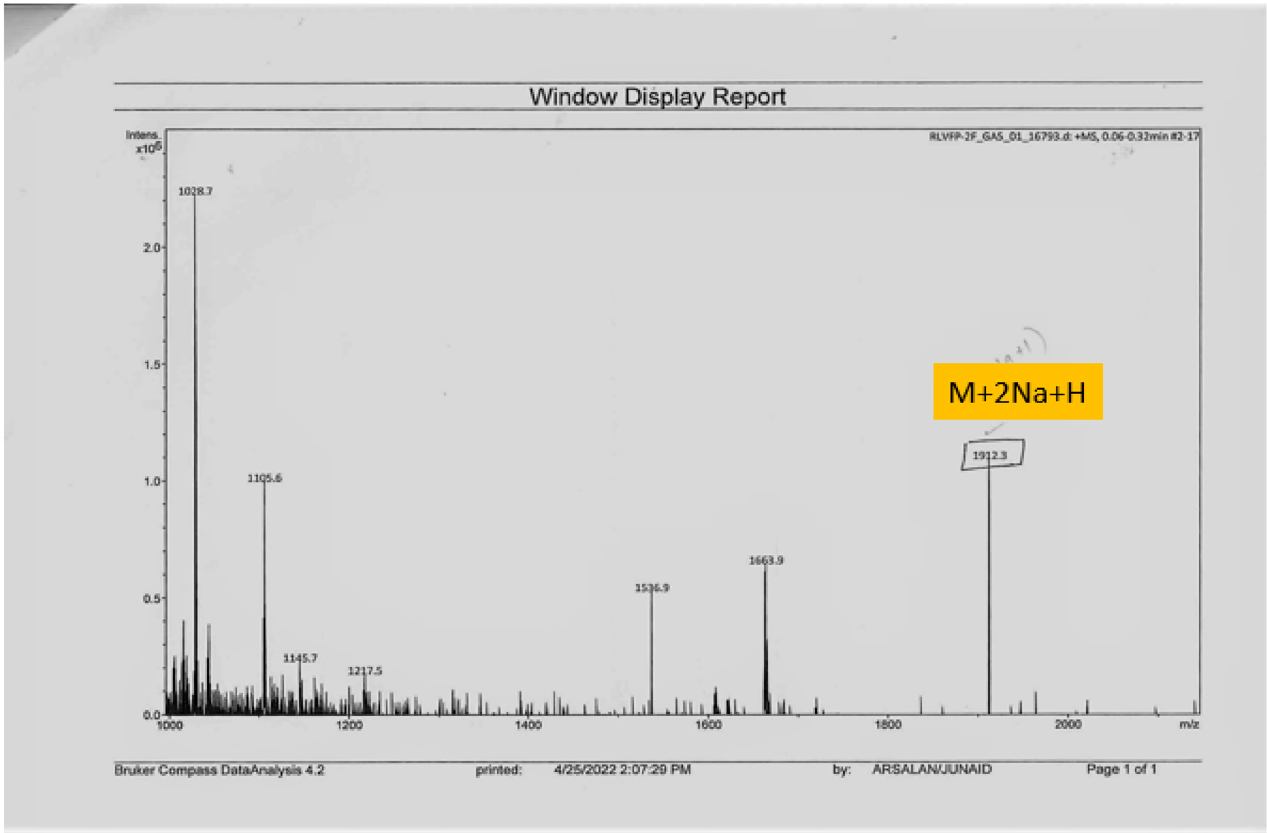

Figure S15: LR-ESI-MS spectrum of RLFP-2 peptide (**5**)

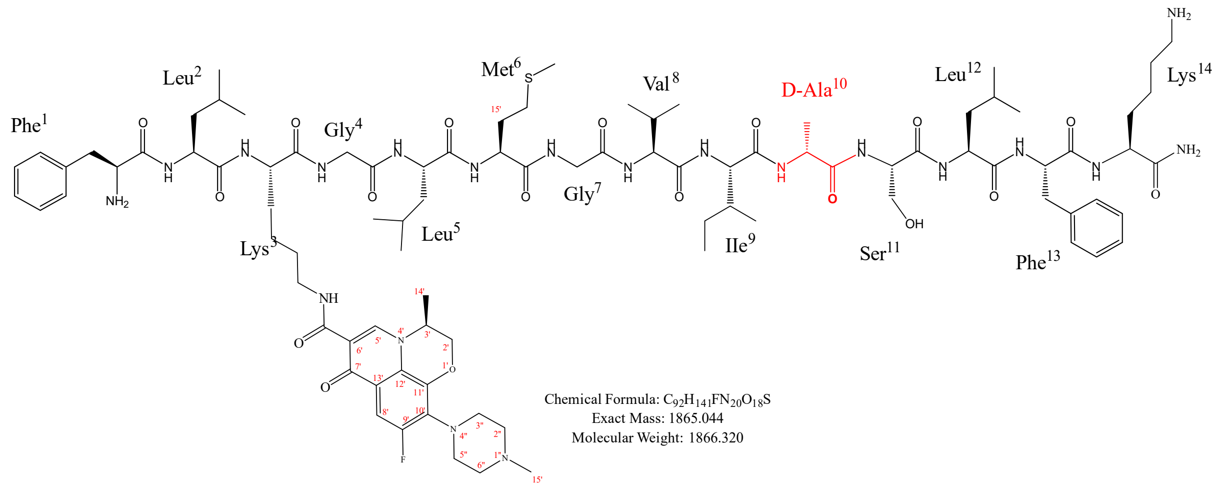

Chemical Formula: $C_{92}H_{141}FN_{20}O_{18}S$
Exact Mass: 1865.044
Molecular Weight: 1866.320

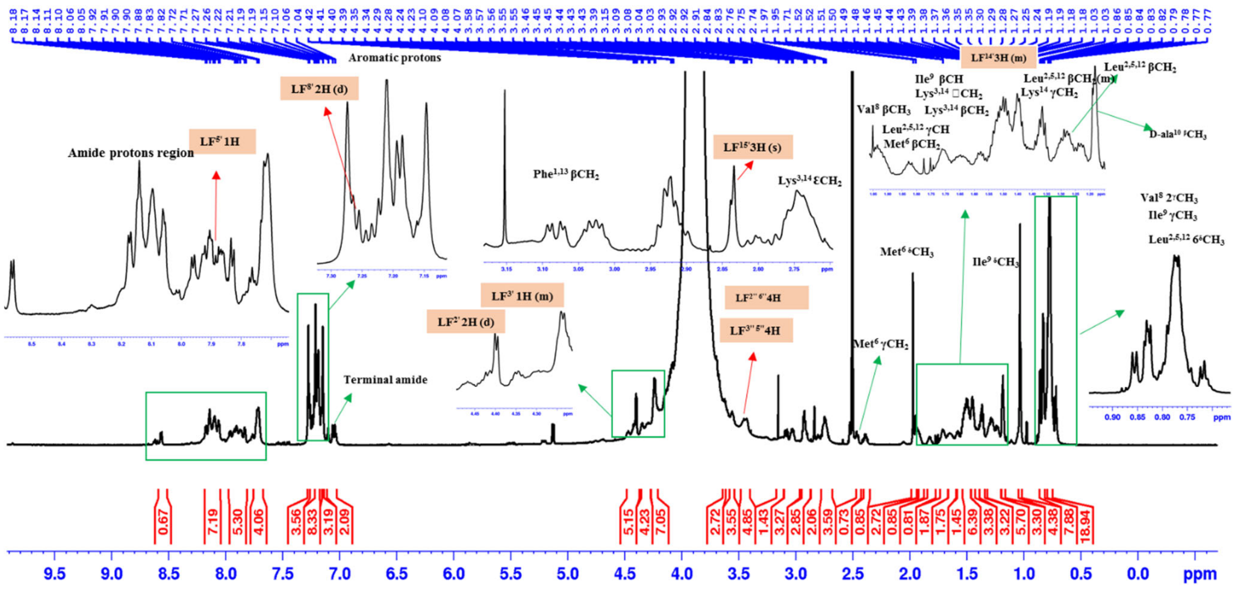

BRUKER

Current Data Parameters
NAME RLVFp-2
EXPNO 1
PROCNO 1

F2 - Acquisition Parameters
Date_ 20221024
Time 9.42 h
INSTRUM spect
PROBHD Z119427_0020 (
PULPROG zg30
TD 65536
SOLVENT DMSO
NS 16
DS 0
SWH 16025.641 Hz
FIDRES 0.489064 Hz
AQ 2.0447233 sec
RG 2.2
DW 31.200 usec
DE 10.00 usec
TE 298.0 K
D1 2.00000000 sec
TD0 1
SFO1 800.2364018 MHz
NUC1 1H
P1 8.00 usec
PLW1 10.94900036 W

F2 - Processing parameters
SI 65536
SF 800.2300063 MHz
WDW EM
SSB 0
LB 0.30 Hz
GB 0
PC 1.00

Figure S16: ^{1}H-NMR spectrum (800 MHz, DMSO-d_6) of RLFP-2 peptide (**5**)

Table S5: NMR (800 MHz, DMSO-d_6) Data of RLFP-2 peptide (**5**)

Residue	Position	RLFP-2 peptide (**5**)		
		**^{1}H-NMR** 600 MHz	***J* (Hz)**	**^{13}C-NMR** 150 MHz
L-Phe[1]	α	4.42 *m*		54.8
	β	3.03, 2.74		37.3
			5.2, 5.04	
	1			137.8
	2-6	(7.21-7.14) *m*		127.0-130.0
	CO			172.3
	NH_2			
L-Leu[2]	α	4.34 *m*		51.7
	β	(1.57, 1.48) *m*		40.3
	γ	(1.48 *m*		24.4
	δ_1CH_3 a	(0.76-0.79) *m*		21.8
	δ_2CH_3 b	(0.77-0.84) *m*		22.0
	CO			172.8
	NH	8.56		
L-lys[3]	α	4.08 *m*		58.3
	β	1.51, 1.63		31.6
	γ	(1.22) *m*		22.6
	δ	1.45		31.6
	ϕ	2.91		40.7
	NH	7.09		

	CO			174.5
	NH			
Levofloxacin	2'	4.29<br>4.46		68.1
	3'	4.68	5.12	54.53
	5'	8.62	7.44	144.8
	6'			116.7
	7' (CO)			169.6
	8'	7.48		103.8
	9'			156.7
	10'			134.0
	11'			141.1
	12'			124.0
	13'			119.7
	14'	1.16		18.3
	15'	3.15 *s*		
	2'', 6''	3.46 *m*		47.0
	3'', 5''	3.43 *m*		54.0
	CO			174.3
Gly[4]	α	(3.68-3.73)		42.5
	CO			169.7
	NH	8.05 *m*		
L-Leu[5]	α	4.23 *m*		51.9
	β	(1.57, 1.48) *m*		40.7

	γ	1.48 *m*		24.5
	δ_1	(0.76-0.79) *m*		21.8
	δ_2	(0.77-0.80) *m*		21.9
	CO			172.9
	NH	7.89		
L-Met[6]	α	4.24 *m*		55.6
	β	(1.81) *m*		31.7
	γ	(2.38-2.45) *m*		29.5
	ϕ CH_3	1.97 *s*		15.0
	CO			172.9
	NH	7.90	7.62	
Gly[7]	α	(3.68-3.77)		42.5
	CO			172.7
	NH	8.13 *m*		
L-Val[8]	α	4.08 *m*		58.7
	β	(1.93) *m*		30.6
	γ1	(0.77) *m*		18.7
	γ2	(0.77) *m*		19.5
	CO			172.3
	NH	(7.86) *m*		
L-Ile[9]	α	4.07 *m*		58.7
	β	(1.71) *m*		36.4
	γ_1 CH_3	(0.75)		15.6
	γ2 CH_2	(1.59) *m*		25.1

	δCH_3	(0.72) *m*	11.3
	CO		170.5
	NH	7.83 *m*	
D-Ala[10]	α	(4.22) *m*	48.9
	β	(1.18) *m*	18.6
	CO		173.2
	NH	7.90 *m*	
L-Ser[11]	α	4.23 *m*	52.5
	β	(3.42-3.45) *m*	61.9
		(3.54-3.57) *m*	
	OH		
	CO		170.6
	NH	8.10	
L-Leu[12]	α	4.21 *m*	52.3
	β	(1.57, 1.48) *m*	40.5
	γ	(1.50) *m*	24.6
	$\gamma 1CH_3$ a	(0.76-0.79) *m*	21.8
	$\gamma 1CH_3$ b	(0.77-0.80) *m*	21.9
	CO		173.2
	NH	78.08 *m*	
L-Phe[13]	α	4.40 *m*	54.7
	β	3.08 *m*	37.3
		2.91 *m*	
	1		137.8
	2-6	(7.21-7.14) *m*	127.0-130.0

	CO		169.6
	NH	7.86	
L-lys[14]	α	4.15 *m*	53.7
	β	1.46, 1.59	31.4
	γ	(1.27) *m*	22.6
	δ	(1.46) *m*	31.8
	ϕ	(2.72-2.77) *m*	39.3
	NH	8.15	
	CO		172.4

Structural Studies of RLFP-3 peptide (**6**)

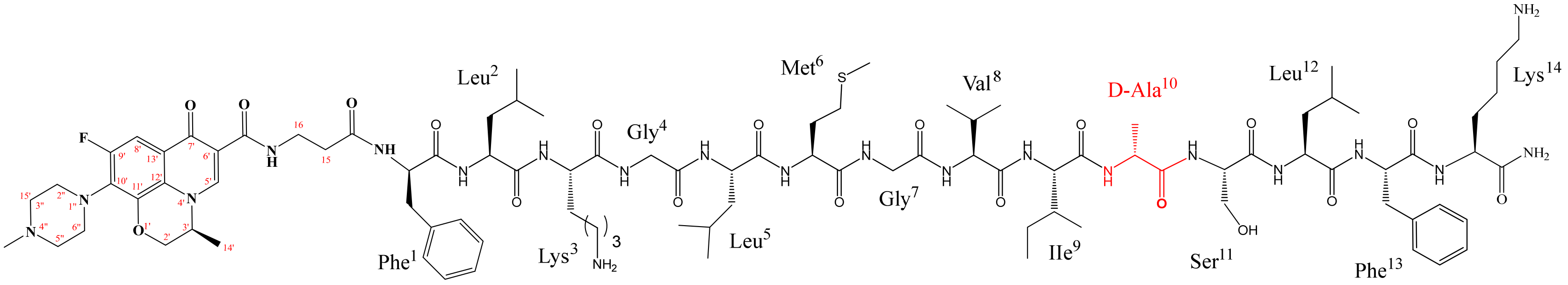

Chemical Formula: $C_{92}H_{141}FN_{20}O_{18}S$
Exact Mass: 1865.044
Molecular Weight: 1866.320

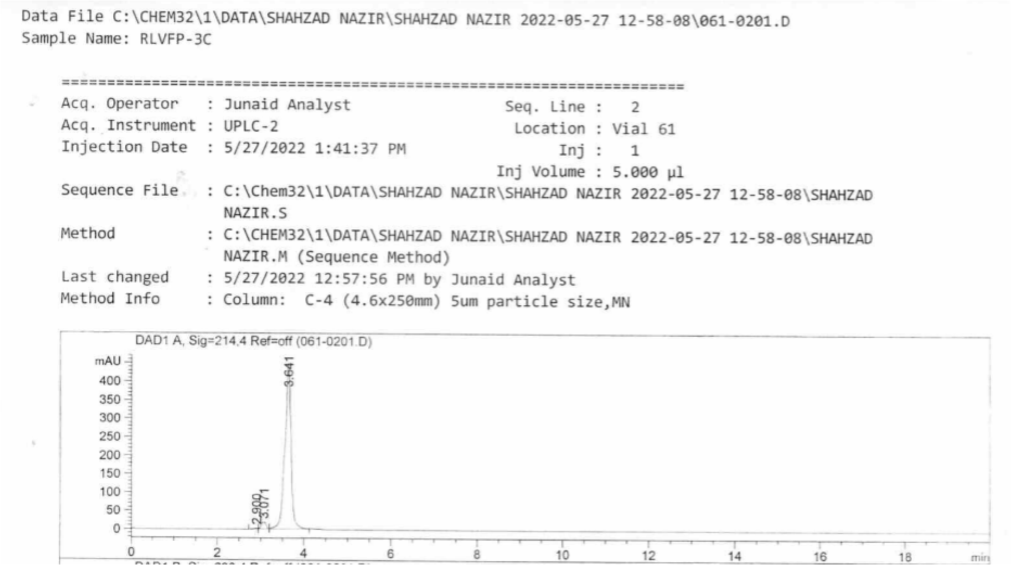

Data File C:\CHEM32\1\DATA\SHAHZAD NAZIR\SHAHZAD NAZIR 2022-05-27 12-58-08\061-0201.D
Sample Name: RLVFP-3C

===

Acq. Operator : Junaid Analyst Seq. Line : 2
Acq. Instrument : UPLC-2 Location : Vial 61
Injection Date : 5/27/2022 1:41:37 PM Inj : 1
Inj Volume : 5.000 µl
Sequence File : C:\Chem32\1\DATA\SHAHZAD NAZIR\SHAHZAD NAZIR 2022-05-27 12-58-08\SHAHZAD NAZIR.S
Method : C:\CHEM32\1\DATA\SHAHZAD NAZIR\SHAHZAD NAZIR 2022-05-27 12-58-08\SHAHZAD NAZIR.M (Sequence Method)
Last changed : 5/27/2022 12:57:56 PM by Junaid Analyst
Method Info : Column: C-4 (4.6x250mm) 5um particle size,MN

Figure S17: UPLC Profile of RLFP-3 peptide (**6**)

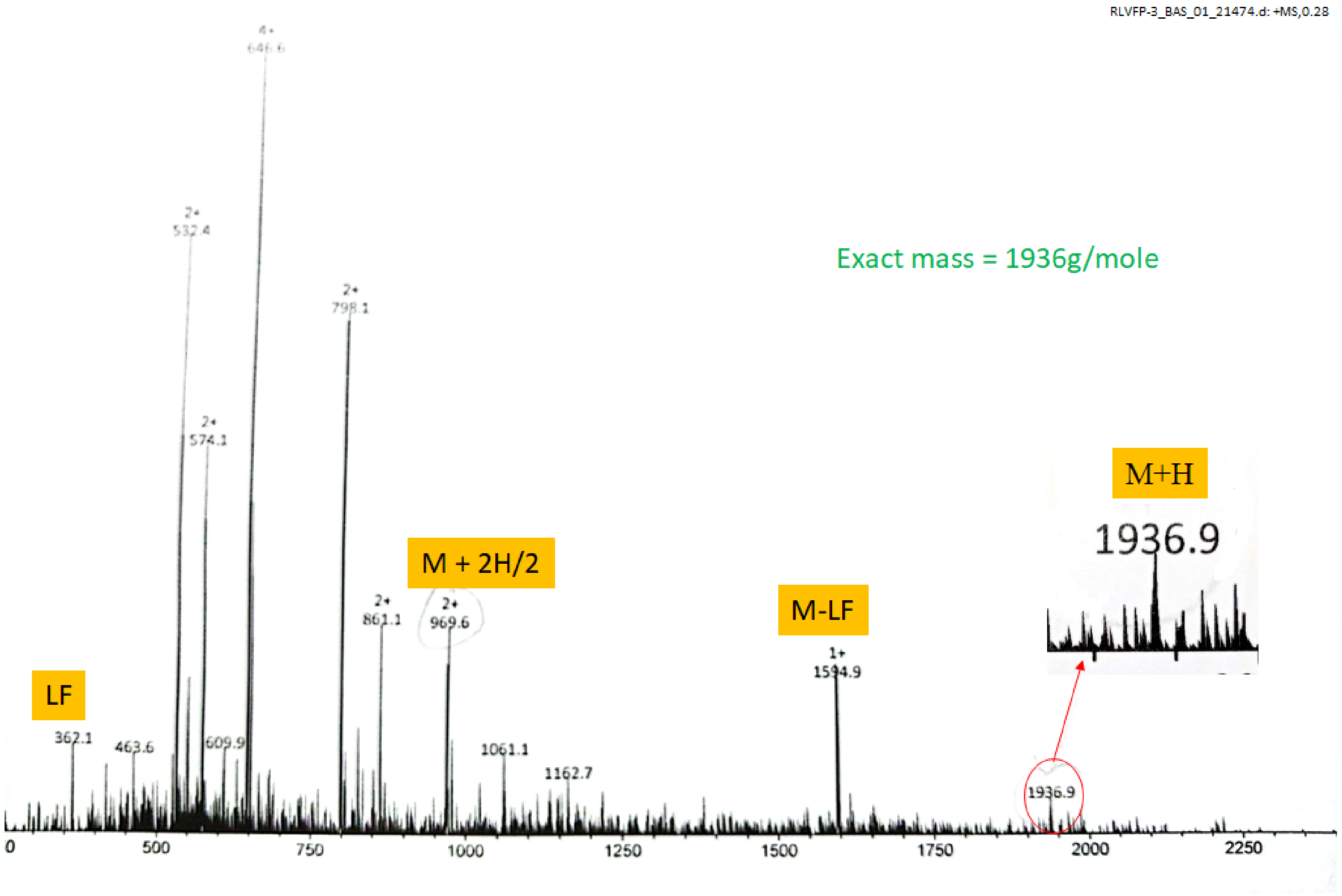

Figure S18: LR-ESI-MS spectrum of RLFP-3 peptide (**6**)

样品名称	RLVFP-3	位置	P1-A2	仪器名称	Instrument 1
用户名称		进样体积	1	进样位置	
样品类型	Sample	IRM 校正状态	成功	数据文件名称	7.d
采集方法	jiachun10%-100%12min.m	注释		采集时间	2024/3/7 8:55:09

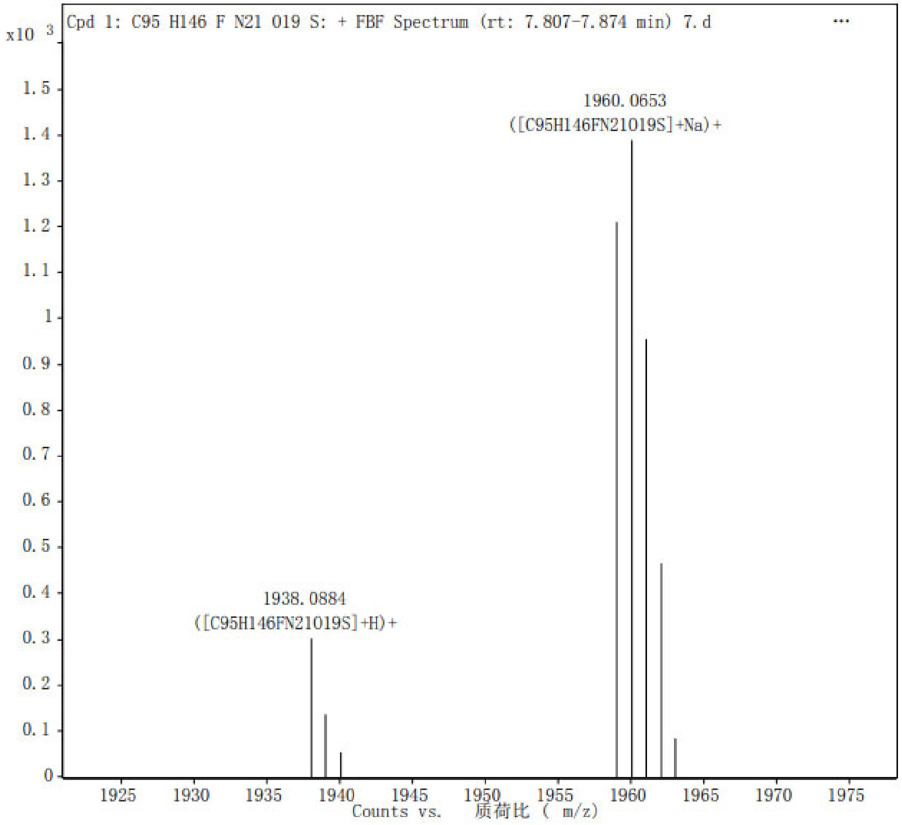

Figure S19: HR-ESI-MS Spectrum of RLFP-3 peptide (**6**)

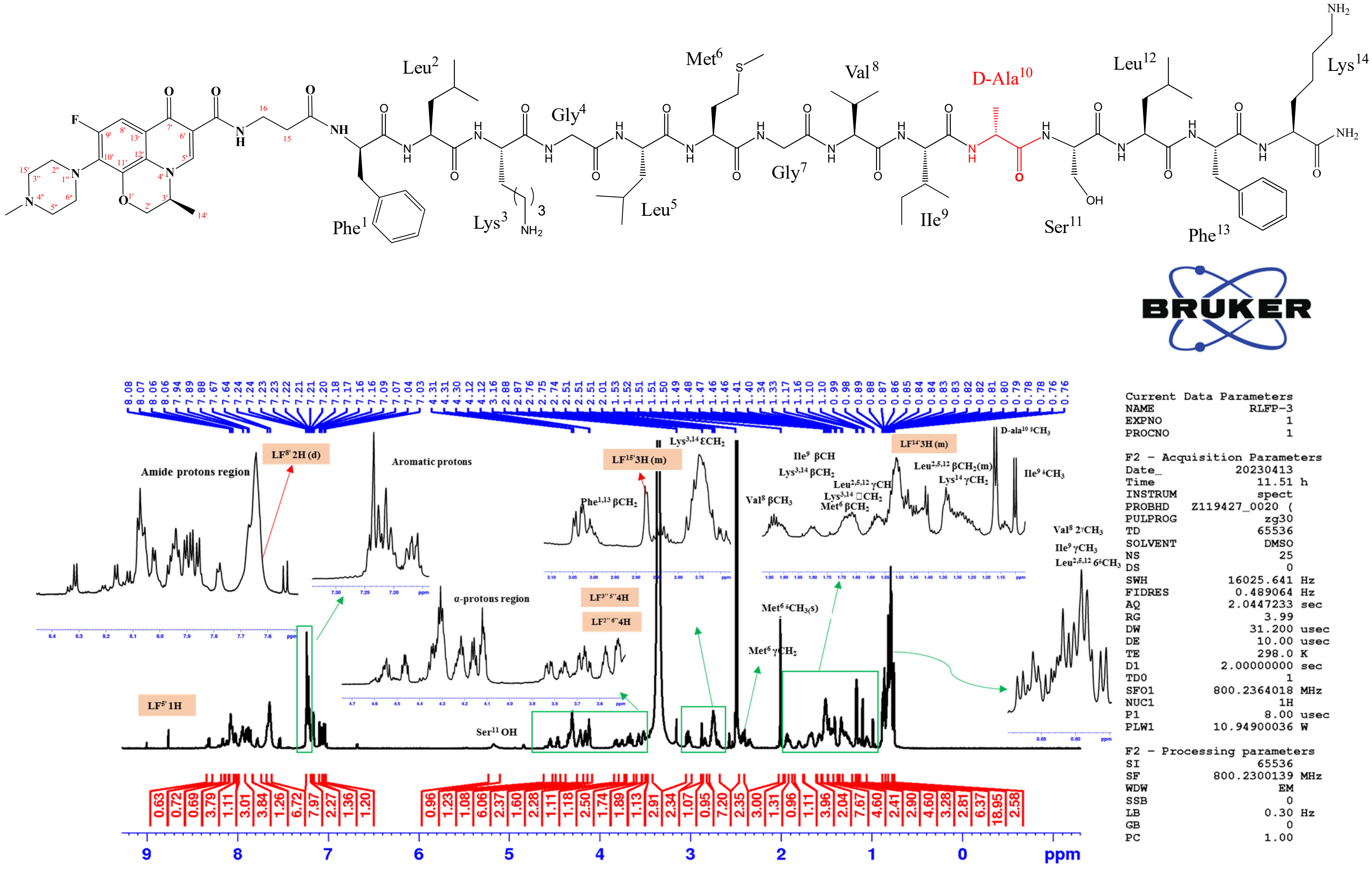

Figure S20: ^{1}H-NMR spectrum (800 MHz, DMSO-d_6) of RLFP-3 peptide (**6**)

Table S6: NMR (800 MHz, DMSO-d_6) Data of RLFP-3 peptide **(6)**

Residue	Position	RLFP-3 peptide (**6**)		
		**^{1}H-NMR** 600 MHz	***J*** **(Hz)**	**^{13}C-NMR** 150 MHz
Levofloxacin	2'	4.31-4.33 *d*	4.62	68.2
		4.54		
	3'	4.84		53.9
	5'	8.76 *s*		145.0
	6'			110.2
	7' (CO)			173.9
	8'	7.52		103.1
	9'			157.5
	10'			129.0
	11'			140.7
	12'			124.2
	13'			122.7
	14'	1.40 *m*		18.06
	15'	2.84 *s*		42.4
	2", 6"	3.16		53.2
	3", 5"	3.47		47.8
	CO			163.8
β-alanine	15	2.34 *m*		35.2
	16	3.44 *m*		53.2
	NH	9.8		
	CO			169.3

L-Phe[1]	α	4.45 *m*		53.7
	β	(2.82-2.85) *m*, (3.15) *s*		37.5
	1			137.5
	2-6	(7.15-7.24) *m*		126.2-129.1
	CO			171.3
	NH	8.31	7.2	
L-Leu[2]	α	4.29		54.5
	β	(1.42-1.45) *m*		40.5
	γ	(1.48-1.54) *m*		24.1
	δ_1CH_3 a	(0.75-0.82) *m*		21.4
	δ_2CH_3 b	(0.85-0.87) *m*		23.2
	CO			171.6
	NH	7.94		
L-lys[3]	α	4.23		52.3
	β	(1.56-1.60) *m*		31.4
	γ	(1.23-1.31) *m*		22.1
	δ	(1.53-1.51) *m*		26.6
	ϕ	(2.71-2.78) *m*		38.7
	NH_2	(7.64-7.66) *bs*		
	CO			171.5
	NH	7.95		
Gly[4]	α	3.6-3.8		41.8
	CO			171.6
	NH	8.06		

L-Leu[5]	α	4.30		54.6
	β	(1.40-147) *m*		40.8
	γ	(1.48-1.50) *m*		24.0
	δ_1	(0.75-0.81) *m*		21.4
	δ_2	(0.83-0.88) *m*		22.9
	CO			170.9
	NH	7.91		
L-Met[6]	α	4.29		51.0
	β	(2.40-2.45) *m*		31.9
	γ	(1.80-1.90) *m*		29.4
	ϕ CH_3	2.00 *s*		14.5
	CO			171.3
	NH	8.05	7.4	
Gly[7]	α	3.6-3.8		41.9
	CO			168.6
	NH	8.06		
L-Val[8]	α	4.21		57.6
	β	(1.92-1.95) *m*		30.5
	γ1	(0.78-0.80) *m*		18.4
	γ2	(0.80-0.83) *m*		19.1
	CO			171.0
	NH	7.78		
L-Ile[9]	α	4.11 *m*		57.3
	β	(1.65-1.69) *m*		36.3

	$\gamma_1 CH_3$	(1.04)		30.5
	$\gamma 2\ CH_2$	(1.60-1.69) *m*		31.5
	δCH_3	(0.78-0.83) *m*		11.2
	CO			171.2
	NH	7.93		
D-Ala[10]	α	4.34		47.9
	β	1.16 *d*	7.22	17.9
	CO			171.9
	NH	8.15		
L-Ser[11]	α	4.31		53.9
	β	(3.50-3.51) *m*		61.6
	OH	5.1 *bs*		
	CO			170.2
	NH	7.88		
L-Leu[12]	α	4.16		51.6
	β	(1.44-1.47) *m*		40.1
	γ	(1.50-1.53) *m*		24.0
	$\gamma 1CH_3$ a	(0.75-0.80) *m*		21.4
	$\gamma 1CH_3$ b	(0.83-0.85) *m*		23.3
	CO			170.3
	NH	7.92		
L-Phe[13]	α	4.46		53.8
	β	2.87-2.94,		37.1
		3.01-3.04 *dd*	4.72, 4.08	
	1			137.8

	2-6	(7.15-7.24) *m*		126.2-129.1
	CO			170.6
	NH	7.85		
L-Lys[14]	α	4.12		52.2
	β	(1.31-1.35) *m*		31.8
	γ	(1.23-1.30) *m*		22.0
	δ	(1.50-1.54) *m*		26.6
	ϕ	2.73-2.78 *m*		38.7
	NH_2	7.03, 7.07		
	CO			173.2
	NH	7.86 *d*	7.94	

Structural Studies of RSP-4 peptide (**7**)

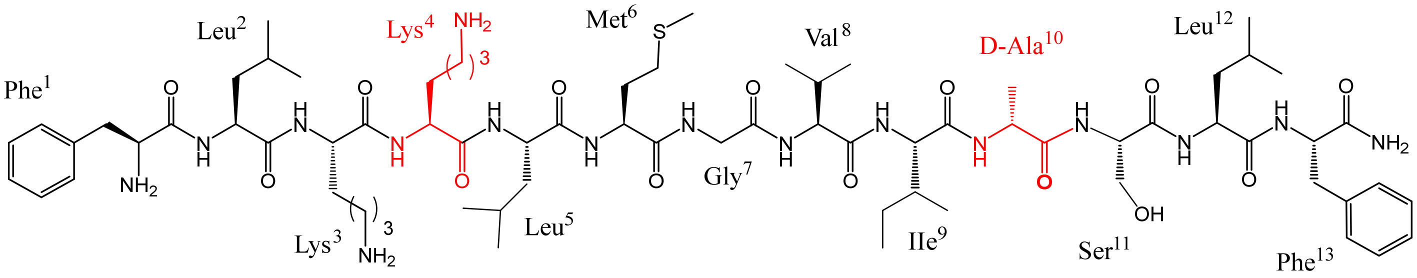

Chemical Formula: $C_{72}H_{120}N_{16}O_{14}S$
Exact Mass: 1464.889
Molecular Weight: 1465.910

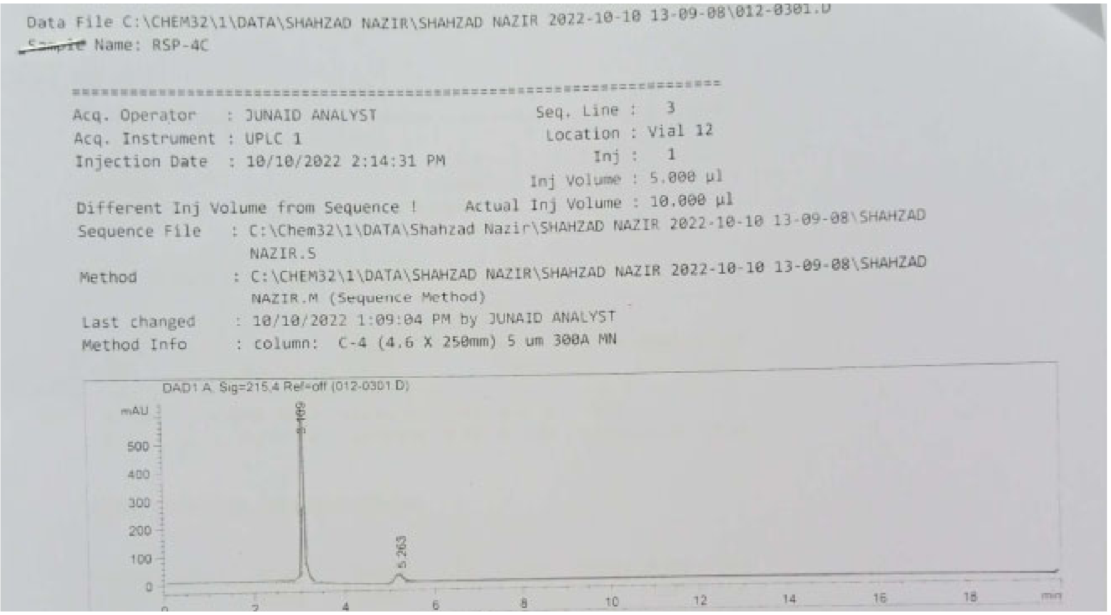

Figure S21: UPLC Profile of RSP-4 peptide (**7**)

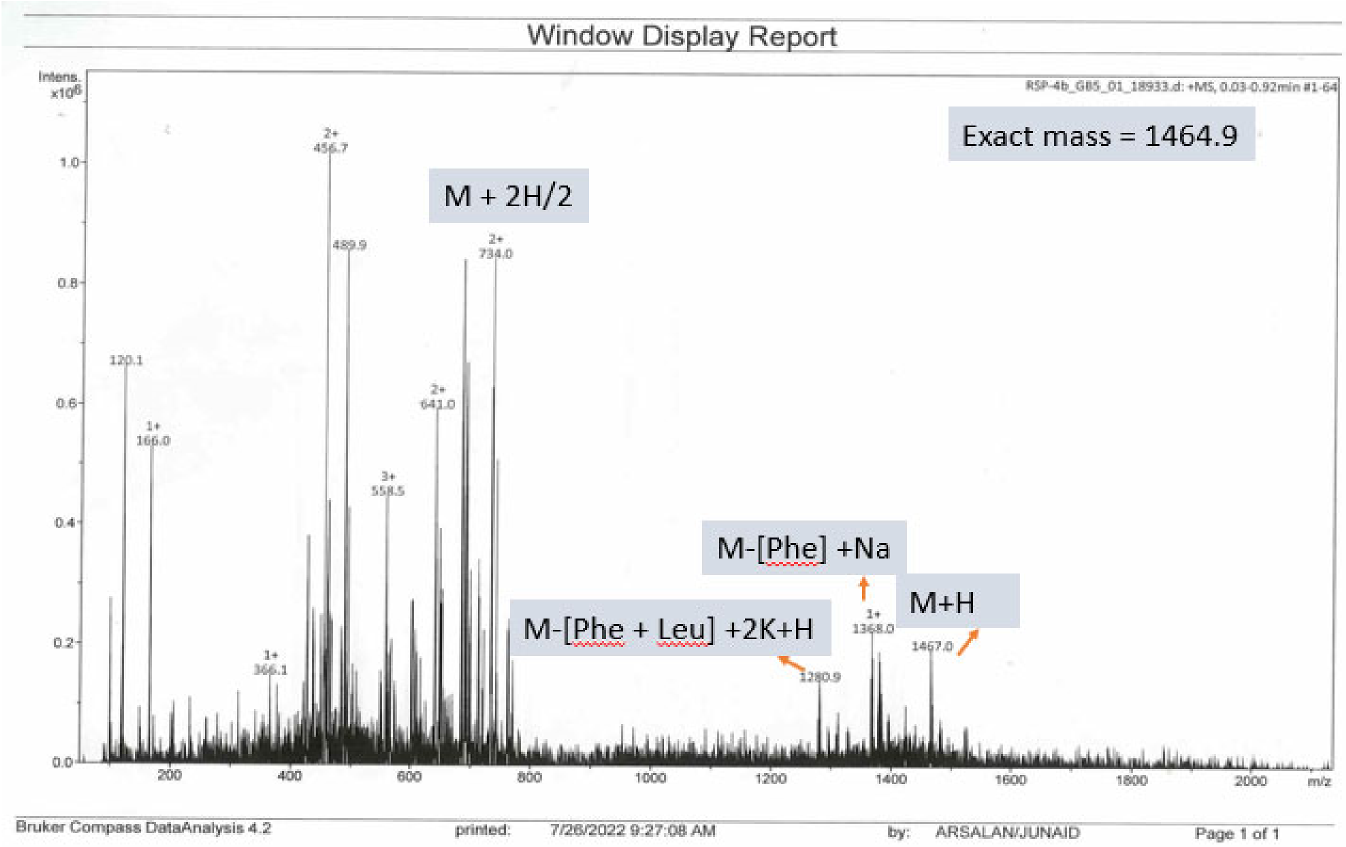

Figure S22: LR-ESI-MS spectrum of RSP-4 peptide (**7**)

样品名称	RSP-4	位置	P1-E3	仪器名称	Instrument 1
用户名称		进样体积	0.8	进样位置	
样品类型	Sample	IRM 校正状态	成功	数据文件名称	RSP-4.d
采集方法	jiachun10%-100%12min.m	注释		采集时间	2024/3/6 13:53:28

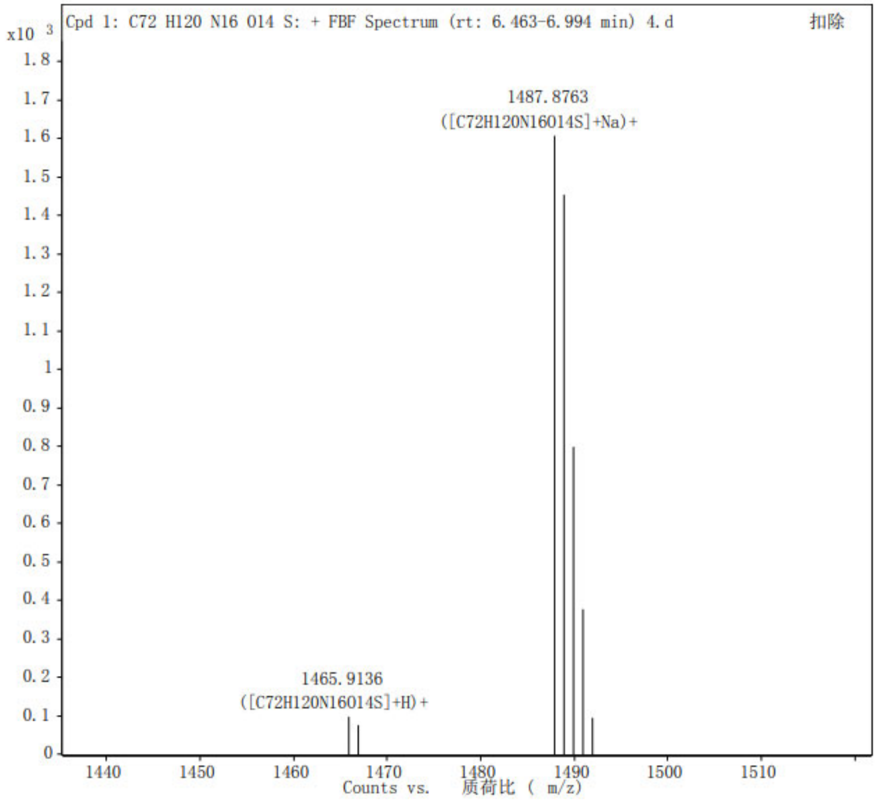

Figure S23: HR-ESI-MS Spectrum of RSP-4 peptide (**7**)

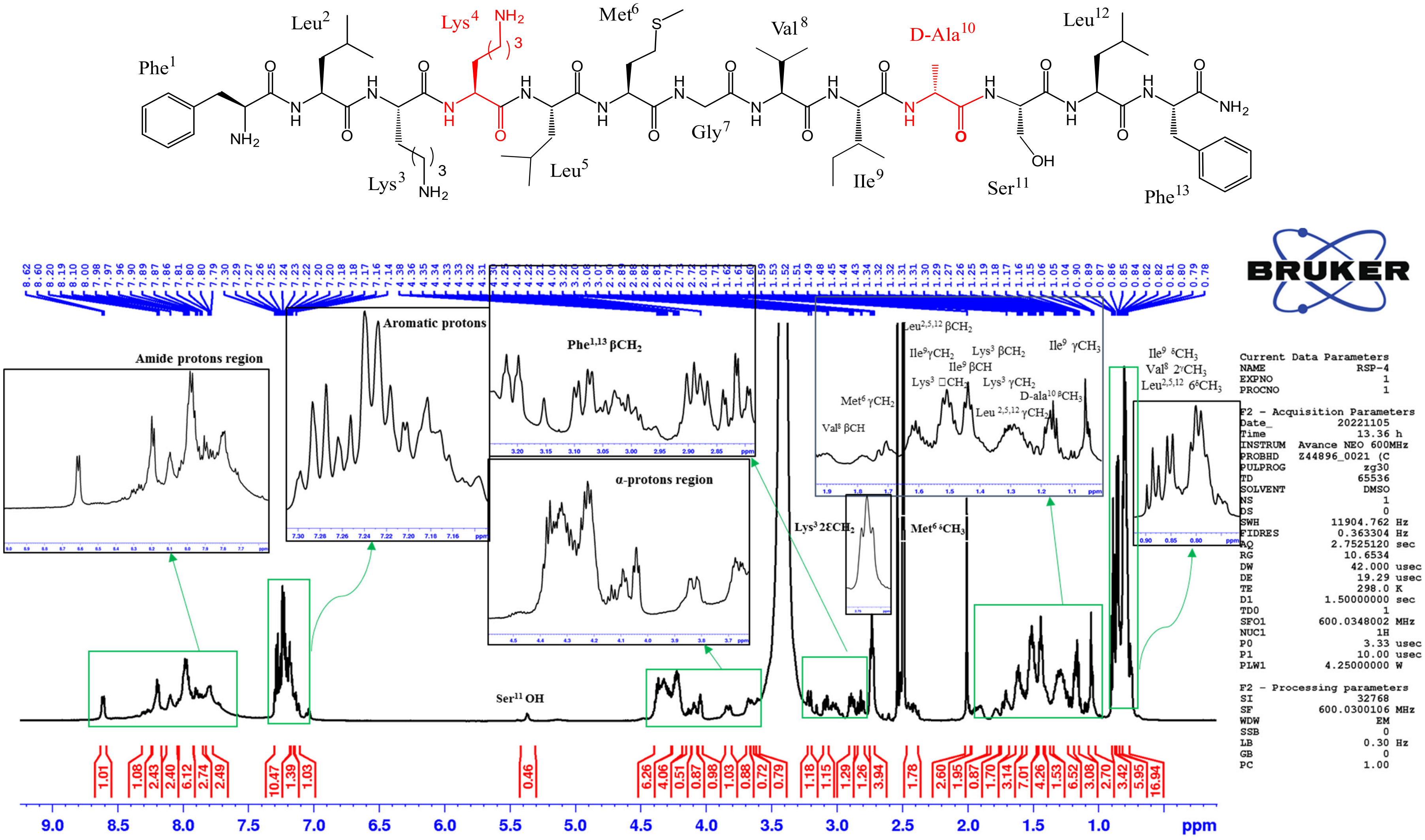

Figure S24: ^{1}H-NMR spectrum (600 MHz, DMSO-d_6) of RSP-4 peptide (**7**)

Table S7-: NMR (600 MHz, DMSO-d_6) Data of RSP-4 peptide (**7**)

**Residue**	**Position**	RSP-4 peptide (**7**)		
		**^{1}H-NMR** 600 MHz	***J*** **(Hz)**	**^{13}C-NMR** 150 MHz
L-Phe[1]	α	4.04 *bs*		53.3
	β	2.89 *d*, 3.03 *m*		37.4
	1			137.9
	2-6	(7.16-7.27) *m*		127.3-128.7
	CO			167.9
	NH_2			
L-Leu[2]	α	4.37 *m*		53.9
	β	(1.29-1.32) *m*		40.4
	γ	(1.45-1.40) *m*		24.0
	δ_1CH_3 a	(0.72-0.81) *m*		21.5
	δ_2CH_3 b	(0.83-0.87) *m*		23.2
	CO			168.7, 171.4
	NH	8.60 *d*		
L-lys[3]	α	4.23 *m*		52.3
	β	(1.43-1.45) *m*		31.6
	γ	1.30 *m*		22.4
	δ	(1.64-1.69) *m*		23.3
	ϕ	2.74 *t*		38.5
	NH_2			
	CO			171.2

	NH	8.19 *m*	
Lys[4]	α	4.22 *m*	57.6
	β	(1.43-1.45) *m*	31.6
	γ	1.30 *m*	22.4
	δ	(1.64-1.69) *m*	23.3
	ϕ	2.74 *t*	38.5
	NH_2		
	CO		171.4
	NH	7.96 *m*	
L-Leu[5]	α	4.09 *m*	51.9
	β	(1.29-132) *m*	41.0
	γ	(1.49-1.45) *m*	24.8
	δ_1	(0.72-0.81) *m*	21.7
	δ_2	(0.83-0.87) *m*	23.1
	CO		171.6
	NH	7.97 *m*	
L-Met[6]	α	4.31 *m*	51.9
	β	2.39 *m*	29.7
	γ	(1.81-1.78) *m*	31.8
	ϕ CH_3	1.99 *s*	15.4
	CO		172.3
	NH	8.01 *m*	
Gly[7]	α	3.80 *m* , 3.77 *m*	41.9
	CO		168.6

	NH	8.09 *m*	
L-Val[8]	α	4.21 *m*	57.6
	β	*1.92 m*	30.6
	γ1	(0.83-0.87) *m*	18.4
	γ2	(0.83-0.87*) m*	19.3
	CO		171.2
	NH	7.79 *m*	
L-Ile[9]	α	4.11 *m*	51.8
	β	(1.45-1.52) *m*	26.8
	γ_1 CH_3	(0.87-0.84) *m*	14.7
	γ2 CH_2	(1.52-1.60) *m*	34.6
	δCH_3	(0.84-0.80) *m*	11.1
	CO		170.8
	NH	7.98 *m*	
D-Ala[10]	α	4.33 *m*	48.2
	β	1.16 *d*	18.3
	CO		172.7
	NH	7.80 *m*	
L-Ser[11]	α	4.32 *m*	52.7
	β	3.54 *m*, 3.60 *m*	61.7
	OH	5.3 *t*	
	CO		171.4, 172.2
	NH	8.10 *m*	
L-Leu[12]	α	4.27 *m*	51.0

	β	(1.43-1.49) *m*	40.8
	γ	(1.55-1.50) *m*	24.2
	$\gamma 1CH_3$ a	(0.73-0.80) *m*	21.6
	$\gamma 1CH_3$ b	(0.80-0.85) *m*	21.7
	CO		171.7
	NH	7.96 *m*	
L-Phe[13]	α	4.39 *m*	51.3
	β	2.87 *m*, 3.15 *m*	37.4
	1		137.9
	2-6	(7.16-7.27) *m*	126.5-129.5
	CO		171.2, 172.4
	NH	7.89 *m*	
	NH_2	7.12 *s*	

Structural Studies of RLFP-4 peptide (**8**)

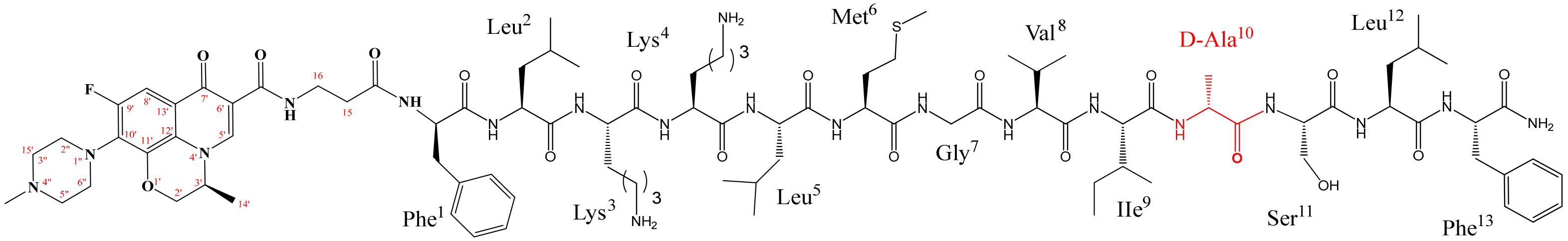

Chemical Formula: $C_{93}H_{143}FN_{20}O_{18}S$
Exact Mass: 1879.059
Molecular Weight: 1880.347

```
=====================================================================
Acq. Operator   : JUNAID ANALYST                    Seq. Line :   3
Acq. Instrument : UPLC 1                             Location : Vial 12
Injection Date  : 3/16/2023 3:59:14 PM                    Inj :   1
                                                   Inj Volume : 5.000 µl
Sequence File   : C:\Chem32\1\DATA\lab 210\LAB 210 2023-03-16 14-53-58\LAB 210.S
Method          : C:\CHEM32\1\DATA\LAB 210\LAB 210 2023-03-16 14-53-58\LAB 210.M (Sequence
                  Method)
Last changed    : 3/16/2023 2:53:54 PM by JUNAID ANALYST
Method Info     : column:  C-4 (4.6 X 250mm) 5 um 300A MN
```

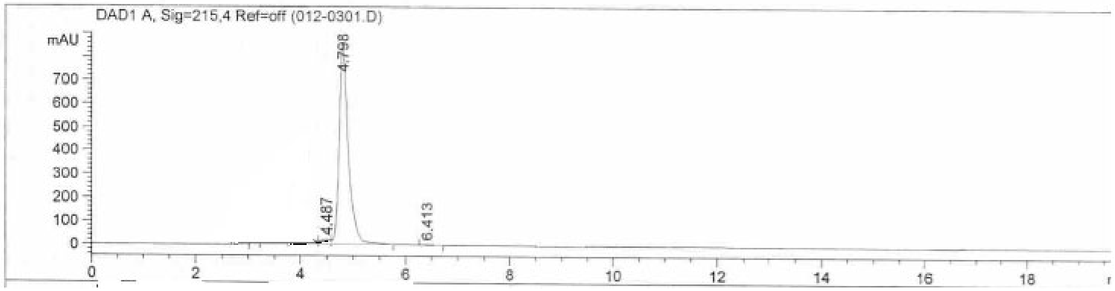

Figure S25: UPLC Profile of RLFP-4-(**8**)

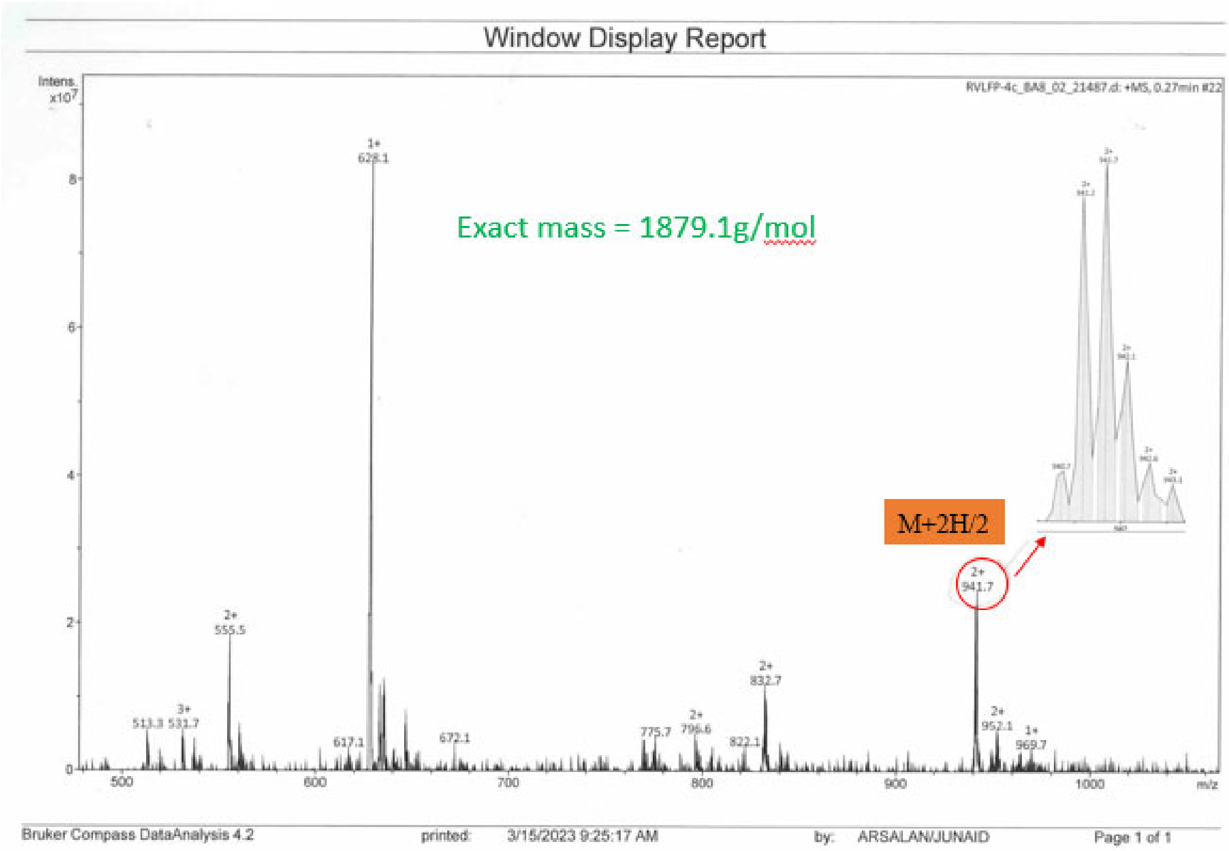

Figure S26: LR-ESI-MS (Low Resolution Positive Mode) spectrum of RLFP-4-**(8)**

样品名称	RLVFP-4	位置	P1-C2	仪器名称	Instrument 1
用户名称		进样体积	1	进样位置	
样品类型	Sample	IRM 校正状态	成功	数据文件名称	8.d
采集方法	jiachun10%-100%14min.m	注释		采集时间	2024/3/8 19:01:53

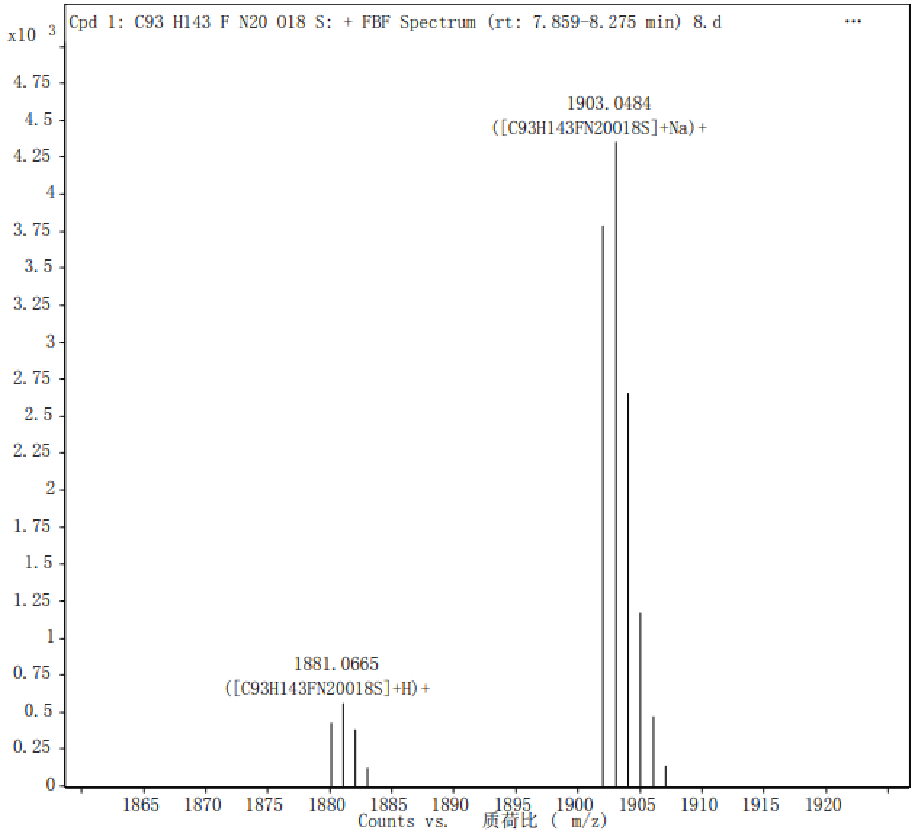

Figure S27: HR-ESI-MS (High Resolution Positive Mode) Spectrum of RLFP-4-**(8)**

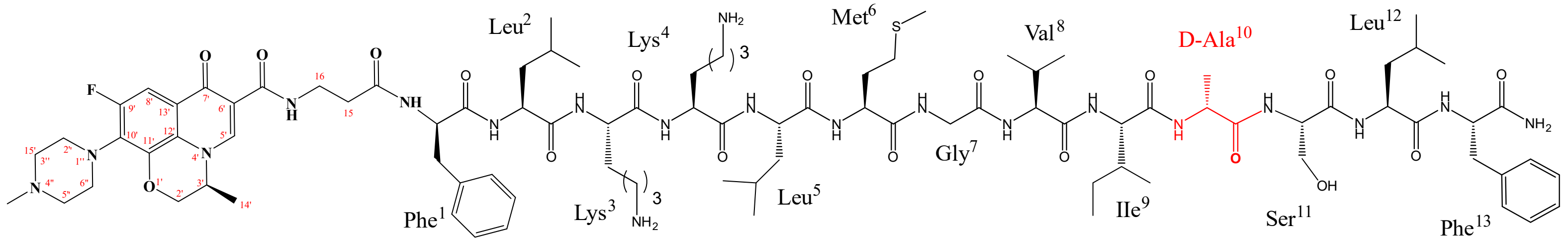

Chemical Formula: $C_{93}H_{143}FN_{20}O_{18}S$
Exact Mass: 1879.059
Molecular Weight: 1880.347

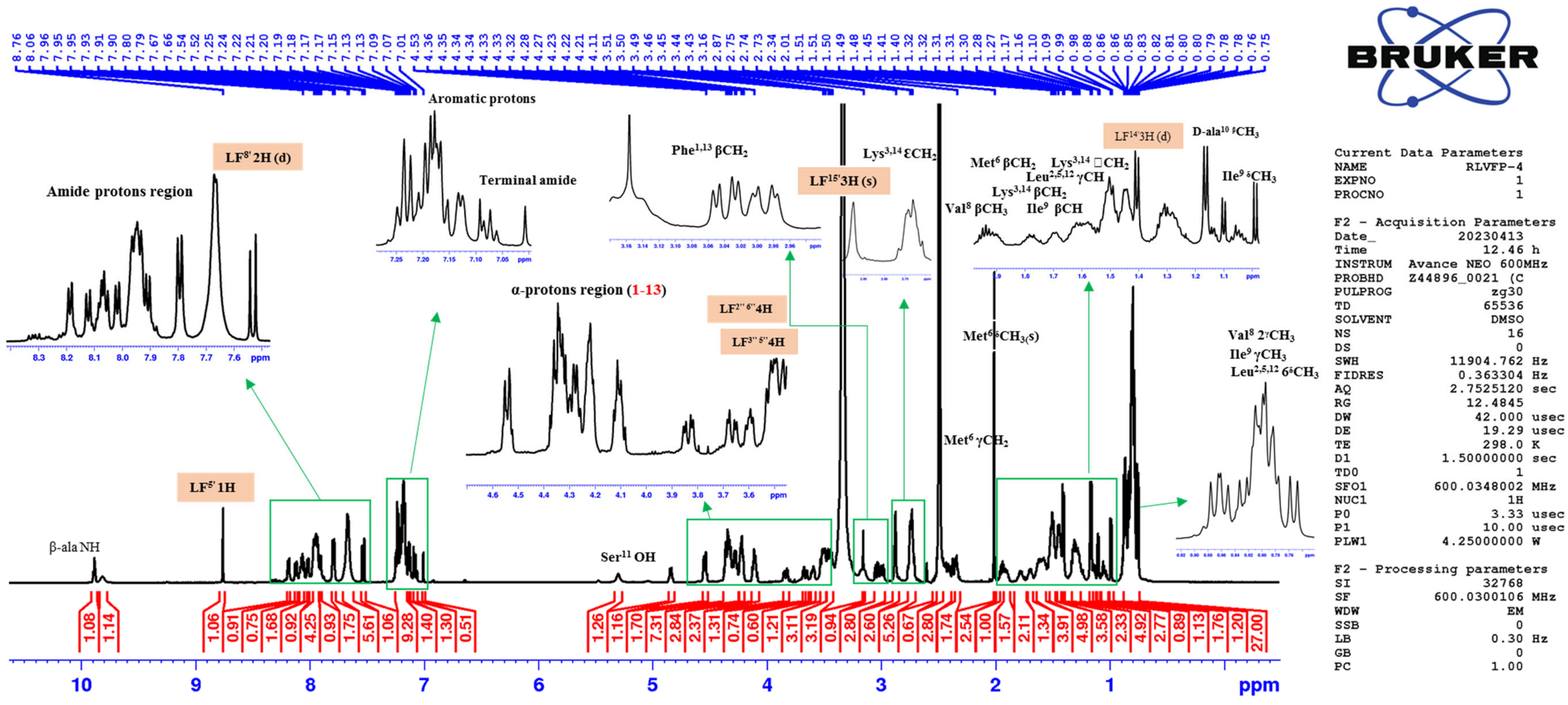

Figure S28: ^{1}H-NMR spectrum (600 MHz, DMSO-d_6) of RLFP-4 peptide (**8**)

Table S8: NMR (600 MHz, DMSO-d_6) Data of RLFP-4 peptide (**8**)

**Residue**	**Position**	RLFP-4 peptide (**8**)		
		**^{1}H-NMR** 600 MHz	***J* (Hz)**	**^{13}C-NMR** 150 MHz
Levofloxacin	2'	4.31-4.33 *d*	4.62	68.2
		4.54		
	3'	4.84		53.9
	5'	8.76 *s*		145.0
	6'			110.2
	7' (CO)			173.9
	8'	7.52		103.1
	9'			157.5
	10'			129.0
	11'			140.7
	12'			124.2
	13'			122.7
	14'	1.40 *m*		18.06
	15'	2.84 *s*		42.4
	2'', 6''	3.16		53.2
	3'', 5''	3.47		53.4
	CO			163.8
β-alanine	15	2.35		35.2
	16	3.37, 3.44		34.9
	CO			170.57
	NH	9.88		

L-Phe[1]	α	4.52 *m*		53.7
	β	(2.87-2.94), (3.02-3.05)		37.2
		dd	4.74, 4.56	
	1			137.7
	2-6	(7.15-7.24) *m*		126.0-129.1
	CO			171.1
	NH	8.12 *d*	8.1	
L-Leu[2]	α	4.26 *m*		51.0
	β	(1.26-1.33) *m*		40.4
	γ	(1.57-1.61) *m*		23.9
	δ_1CH_3 a	(0.74) *d*	6.54	21.3
	δ_2CH_3 b	(0.78-0.80) *m*		22.9
	CO			171.9
	NH	8.07		
L-lys[3]	α	4.23 *m*		51.9
	β	(1.57-1.60) *m*		32.03
	γ	1.45 *m*		26.6
	δ	(1.26-1.33) *m*		22.1
	ϕ	2.70-2.76 *m*		38.7
	NH_2			
	CO			171.1
	NH	7.94 *d*	12.18	
L-Lys[4]	α	4.22 *m*		51.9
	β	(1.57-1.61) *m*		31.2

	γ	1.45 *m*		26.6
	δ	(1.26-1.33) *m*		22.1
	ϕ	2.70-2.76 *m*		38.7
	NH_2			
	CO			171.0
	NH	7.80 *m*	4.5	
L-Leu[5]	α	4.09 *m*		52.1
	β	(1.33-137) *m*		40.4
	γ	(1.43-1.46) *m*		24.08
	δ_1	(0.77-0.80) *m*		21.4
	δ_2	(0.80-0.83) *m*		23.2
	CO			171.4
	NH	8.18		
L-Met[6]	α	4.32 *m*		53.9
	β	(2.41-2.45) *m*		32.0
	γ	(1.80-1.90) *m*		29.3
	ϕ CH_3	2.00 *s*		15.1
	CO			170.4
	NH	8.03 *d*	7.62	
Gly[7]	α	(3.66)<br>(3.84) *m*		41.8
	CO			168.4
	NH	8.08 *m*		
L-Val[8]	α	4.30 *m*		57.5

	β	(1.89-1.96) *m*		30.5
	γ1	(0.78-0.80) *m*		18.0
	γ2	(0.80-0.83) *m*		17.9
	CO			171.6
	NH	7.95 *m*		
L-Ile[9]	α	4.11 *m*		52.1
	β	(1.79-1.82) *m*		23.2
	$\gamma_1 CH_3$	(1.10) *d*	4.74	22.2
	γ_2 CH_2	(1.04-1.06) *m*		24.5
	δCH_3	(0.75-0.78) *m*		10.9
	CO			171.2
	NH	7.97 *m*		
D-Ala[10]	α	4.33 *m*		47.9
	β	1.16 *d*	6.96	18.4
	CO			170.5
	NH	7.92 *m*		
L-Ser[11]	α	4.35 *m*		53.3
	β	(3.61-3.68) *dd*	5.22, 5.16	61.7
		(3.81-3.85)*dd*	5.64, 5.82	
	OH	5.3 *bs*		
	CO			171.6
	NH	8.02		
L-Leu[12]	α	4.13 *m*		54.4
	β	(1.33-1.37) *m*		40.6
	γ	(1.47-1.57) *m*		24.5

	γ1CH_3 a	(0.75-0.80) *m*		21.3
	γ1CH_3 b	(0.83-0.85) *m*		23.0
	CO			170.6
	NH	7.96 *m*		
L-Phe[13]	α	4.35 *m*		51.1
	β	2.87-2.94,		37.2
		3.01-3.10 *d*	4.74	
	1			137.8
	2-6	(7.00-7.30) *m*		126.0-129.0
	CO			172.5
	NH	8.09		
	NH_2	7.79		

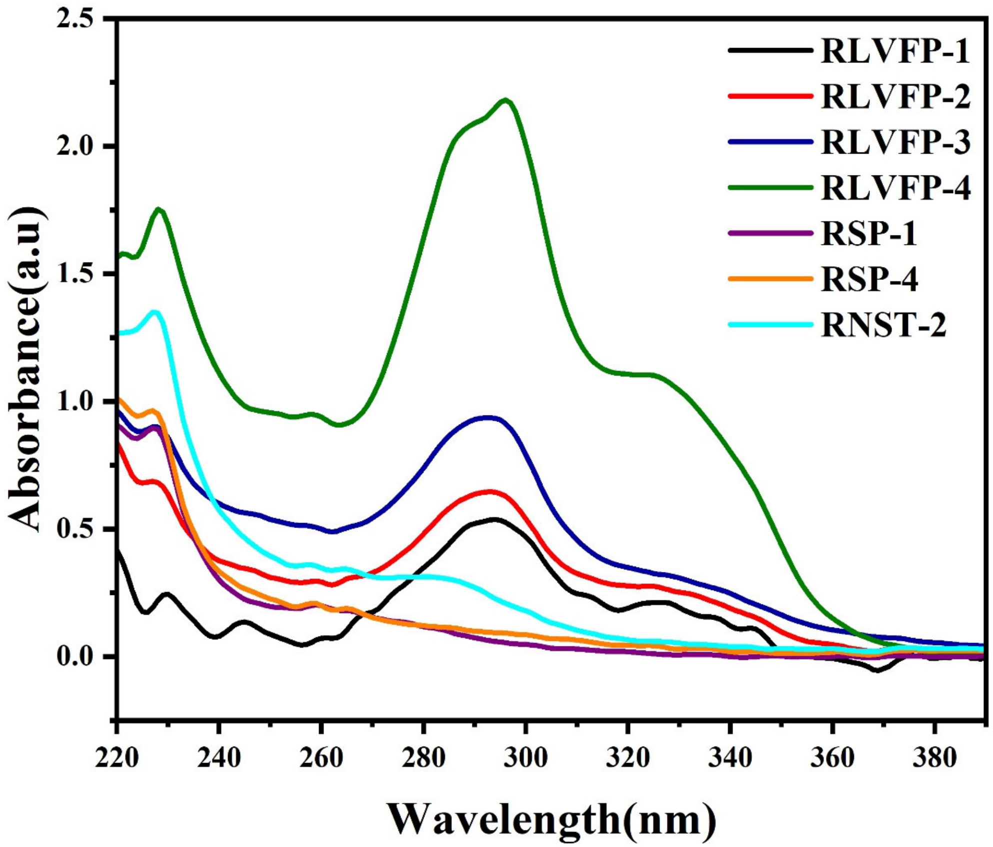

Figure S29: UV-Vis spectra (MeOH) of retro analogues RNST-2-(**2**), RSP-1 peptide (**3**), RSP-4 peptide (**7**), RLFP-1 peptide (**4**), RLFP-2 peptide (**5**), RLFP-3 peptide (**6**) and RLFP-4**-** peptide (**8**)

References

1. Shaheen F, Nadeem-ul-Haque M, Ahmed A, Simjee SU, Ganesan A, Jabeen A, Shah Z.A. Choudhary, M.I. Synthesis of breast cancer targeting conjugate of temporin-SHa analog and its effect on pro-and anti-apoptotic protein expression in MCF-7 cells. *Peptides* 2018. **106**: p. 68-82.
